# The Toxic Effects of Antibiotics on Freshwater and Marine Photosynthetic Microorganisms: State of the Art

**DOI:** 10.3390/plants10030591

**Published:** 2021-03-21

**Authors:** Lilianna Sharma, Grzegorz Siedlewicz, Ksenia Pazdro

**Affiliations:** Department of Marine Chemistry and Biochemistry, Institute of Oceanology, Polish Academy of Sciences, 81-712 Sopot, Poland; gsiedlewicz@iopan.gda.pl (G.S.); pazdro@iopan.gda.pl (K.P.)

**Keywords:** antibiotics, photosynthesis, oxidative stress, nontarget, marine microorganisms

## Abstract

Antibiotic residues have been commonly detected worldwide in freshwater, estuarine, and marine ecosystems. The review summarizes the up-to-date information about the toxic effects of over 60 antibiotics on nontarget autotrophic microorganisms with a particular focus on marine microalgae. A comprehensive overview of the available reports led to the identification of significant knowledge gaps. The data on just one species of freshwater green algae (*Raphidocelis subcapitata*) constitute 60% of the total information on the toxicity of antibiotics, while data on marine species account for less than 14% of the reports. Moreover, there is a clear knowledge gap regarding the chronic effects of antibiotic exposure (only 9% of studies represent exposition time values longer than 7 days). The review summarizes the information on different physiological endpoints, including processes involved in photosynthesis, photoprotective and antioxidant mechanisms. Currently, the hazard assessment is mostly based on the results of the evaluation of individual chemicals and acute toxicity tests of freshwater organisms. Future research trends should involve chronic effect studies incorporating sensitive endpoints with the application of environmentally relevant concentrations, as well as studies on the mixture effects and combined environmental factors influencing toxicity.

## 1. Introduction

Pharmaceuticals are one of the groups of emerging pollutants present in ground and surface waters, soils, and sediments. Unlike many other contaminants, they are continuously released into the environment at low concentrations, contributing to the rise in their overall toxicity and so-called pseudo-persistency. Pharmaceuticals are specially contrived to induce specific biological effects and resist inactivation. Paradoxically, the same properties are accountable for their toxicity in terrestrial and aquatic ecosystems. A significant portion of administered human and veterinary pharmaceuticals are largely unmetabolized and therefore excreted as unaltered parent compounds or their active metabolites. Sources of aquatic contamination include wastewater effluents, illegal and uncontrolled medicine disposal, aquacultures, manure application, and surface run-off [1,2]. Once in the environment, pharmaceutical substances undergo degradation through the main abiotic pathways: biodegradation, hydrolysis, and photolysis. The concentration of pharmaceuticals or more precisely active pharmaceutical ingredients (APIs) generally ranges between ng/L to low µg/L in the surface waters and µg/g to ng/g in sediments [3]. It correlates with the volume of the receiving body of water, the density of the human population in the drainage basin, and technologies used in wastewater treatment [4,5]. Thus far, the occurrence of pharmaceuticals in the marine environment—the sink of the continental contamination—has been less explored due to significant dilution and complexity of the matrix [6]. Thousands of APIs are currently available on the market with over 600 pharmaceutical contaminants known worldwide [7]. Therefore, the identification of priority substances of the highest hazard to organisms is of great significance. The prioritization approach can be based on persistence, detection levels of the environmental compartments, and the probability of exceeding an effective concentration following the sales of the pharmaceuticals.

Antimicrobial agents have been ranked amongst the substances of principle concern [8]. The consumption of antibiotics has been growing globally, with an estimated 65% increase between the years 2000 and 2015 and a 200% projected global consumption growth for the year 2030 [9]. Antibiotics are broadly used in the human and veterinary treatment of bacterial infections and aquacultures. They are mostly hydrophilic substances of low biodegradability, thus are particularly mobile in aquatic compartments. Several classes of antibiotics have been commonly detected worldwide in freshwater, estuarine, and marine ecosystems [6,10,11,12]. Thus far, amoxicillin, ciprofloxacin, and a total of three representatives of macrolide antibiotics (azithromycin, erythromycin, and clarithromycin) have been listed on the EU Watch List of emerging pollutants (Decision EU 2015/495) [13]. Antibiotics possess inherent biological activity and are continuously introduced into the aquatic environment, and thus pose an imminent threat to nontarget microalgae, whose organelles, akin to mitochondria and chloroplasts, possess structural and evolutionary similarities to bacteria [14]. Studies show high differences in responses of different algal species to antibiotics that can be attributed to several potential factors such as presence and differences in efflux pumps used in the removal of toxic substances within the cell, the enzymatic inactivation as a result of modification or degradation of antibiotics’ structure. Other factors include the bioavailability of the given compound based on the pH of the medium and pKa value and the properties of the binding sites of different algal species [15]. Generally, the mechanisms of action of antibiotics against bacterial biomolecules include inhibition of protein, cell wall, and nucleic acid synthesis, antimetabolite activity, and cell membrane modification [16]. Some reports show that antibiotics can adversely influence the photosynthesis of autotrophic organisms, including both eukaryotes and prokaryotes [17,18]. The probable mechanism of action of some antibiotics such as, e.g., quinolones, towards cyanobacteria is based on the disruption of DNA replication or protein synthesis, while in green algae it could be associated with inhibition of the photosynthetic processes resulting in growth suppression [2,19].

Basic, standard parameters applied in the process of the assessment of the toxic effects are the median effective concentration (EC_50_) or median lethal concentration (LC_50_). Typically, these values are calculated based on growth suppression. The exposure times of classical ecotoxicity tests OECD do not exceed 72h/96h and the test focuses on the growth inhibition of the recommended types of cyanobacteria [20]. A part of reported EC_50_ values of drug exposure hence represents data on just two species of cyanobacteria: marine *Synechococcus leopoliensis* and freshwater *Anabaena flosaquae* [18]. According to the EU Directive 93/67/EEC, the compounds are classified based on the EC_50_ value towards aquatic organisms. Substances with EC_50_ values ranging from 10 to 100 mg/L, 1 to 10 mg/L, and lower than 1 mg/L are considered harmful, toxic, and very toxic, respectively. Compounds with EC_50_ values above 100 mg/L are not classified [21]. In environmental risk assessments, the median effective (EC_50_), the lowest observed effect (LOEC), and no observed effect (NOEC) concentrations are used to calculate and predict the concentration of a substance below which the occurrence of the adverse effect is not likely. A risk quotient of predicted environmental concentration (PEC) and predicted no-effect concentration (PNEC) is calculated. The ratio exceeding or equal to 1 means possible risk to the ecosystem [22]. Some of the later studies focus on diverse effects such as photosynthesis inhibition, oxidative stress defense, proteomic responses, or microcystin synthesis [23,24,25,26,27]. On one hand, the differences in the selection of endpoints and exposure times can lead to inconsistencies in the obtained EC_50_ values for the given compounds. On the other hand, incorporating various endpoints with emphasis on physiological processes such as photosynthesis rather than growth and biomass inhibition accompanied by prolonged exposure times could provide more information about modes of action and reflect the real environmental risks. Comparatively less data are available regarding the chronic effects of antibiotic exposure as most of the ecotoxicological studies focus on acute effects [17,27]. Moreover, the majority of studies and standardized tests have focused on just several species of mainly freshwater green algae and cyanobacteria, with less attention being paid to marine microorganisms. The negative influence of antibiotics on marine microorganisms has been observed even at concentrations as low as ppt (ng/L) and ppb (µg/L) [28]. The sensitivity of algae towards antibiotics is greater compared to daphnia and fish species with median effective concentrations (EC_50_) oscillating at the mg/L range for chlorophytes and µg/L range for cyanobacteria. Relatively less data are available regarding diatoms with the reported literature values at the mg/L level [29].

Currently, the regulatory risk and hazard assessment are mostly based on the evaluation of individual chemicals. Since ecosystems are exposed to multicomponent pharmaceutical mixtures, the possible synergistic, antagonistic, and additive nature of the combined effects of chemicals ought to be adequately addressed. The evaluation of toxicity of combination mixtures as a part of reliable and accurate risk assessment is a very complex and daunting task yet is vital to predicting the possible unintended effects on ecosystems and aquatic wildlife [30].

The article aims to comprehensively present the available information on the impact of antibiotic residues and their mixtures on autotrophic microorganisms with emphasis on marine green algae and cyanobacteria including information about the potential underlying modes of action based on the assessment of the adverse effects on the processes involved in the photosynthesis and photoprotective and antioxidant mechanisms.

## 2. Summary of Available Toxicity Data on Individual Antibiotics

### 2.1. Biomass Endpoint Assessment

**Figure 1:**
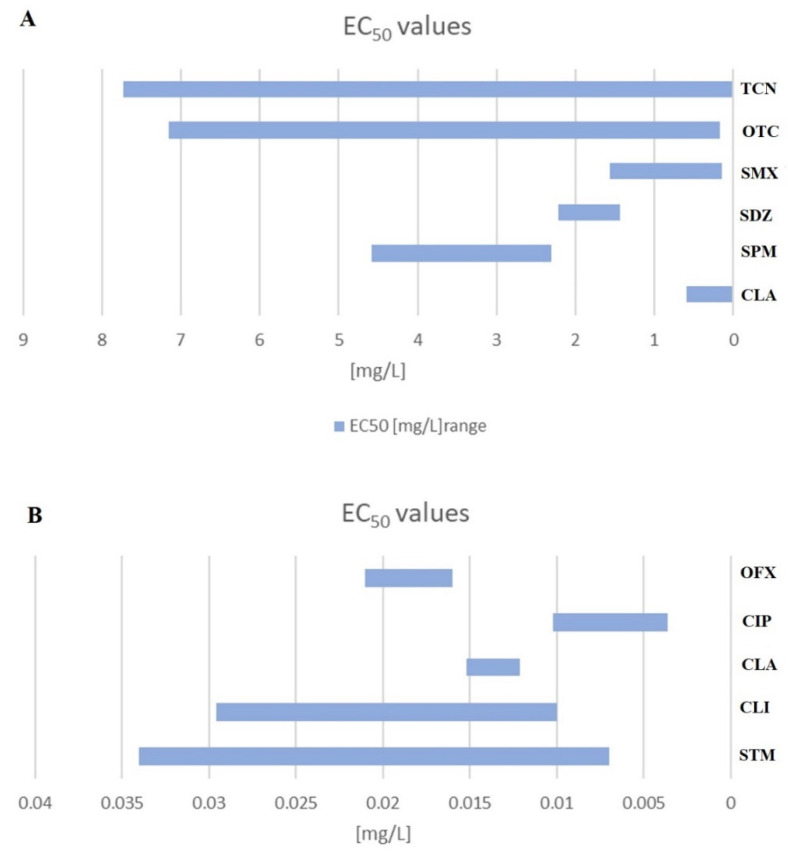
The summary of the EC_50_ values expressed in mg/L of the ten most toxic antibiotics towards freshwater and marine green algae (**A**) and cyanobacteria (**B**) (detailed information can be found in Table S1). (CLA—clarithromycin, CLI—clindamycin, CIP—ciprofloxacin, OFX—ofloxacin, OTC—oxytetracycline, SDZ—sulfadiazine, SMX—sulfamethoxazole, SPM—spiramycin, STM—streptomycin, TCN—tetracycline).

Algae and cyanobacteria form the base of the food chain. Therefore, shifts in their populations can affect the balance of the whole ecosystem as a consequence of the disruption of their important functions including, biogeochemical and nutrient cycling. Cyanobacteria represent a big proportion of phytoplankton mass and are largely responsible for carbon dioxide, nitrogen fixation, and free oxygen production [23,31]. Diatoms present in marine and freshwater environments possess siliceous cell walls and contribute to the production of 25% of world oxygen and 40% of primary marine production [32]. Hence, algae are routinely applied as indicator organisms in the risk assessment studies of human and veterinary pharmaceuticals. They are characterized by quick response times and high sensitivity. As a result of the susceptibility of green and blue algae to antibiotics, cyanobacteria are also included as primary test species in the authorized process of environmental risk assessment [21,33]. Table S1 summarizes the available toxicity data on the effects of a total of 63 antibiotics and their degradation products on nontarget, autotrophic marine, and freshwater microorganisms (green algae, cyanobacteria, and diatom). The toxic effects of sixteen antibiotic classes including aminoglycosides, beta-lactams, tetracyclines, and quinolones presented as median effective concentrations (EC_50_) are based mainly on biomass endpoints, including growth inhibition and cell density. The most studied antibiotic group based on the number of records regarding EC_50_ values is quinolones with 78 records, followed by tetracyclines (55 records), macrolides (58), sulphonamides (44), amphenicols (27), diaminopyrimidine and beta-lactams (26 and 27), lincosamides (15), aminoglycosides (9), quinoxalines and nitroimidazoles (3), pleuromutilins and cephalosporins (2), rifamycins (1), and oxazolidinones (1). The most studied antibiotic is oxytetracycline with 29 records on the median effective toxicity towards green algae (four), cyanobacteria (nine), and diatom (one), respectively (followed by trimethoprim (26 records), erythromycin (21), and enrofloxacin, tylosin and sulfamethoxazole (15)). The EC_50_ values obtained in the toxicity tests exceeding the standard exposure time (≥96 h) constitute only 31% of records (110 out of a total 352), but only 9% (32) represent values of an exposition time longer than 7 days. The toxicity of the different antibiotics within the same group varies towards microorganism species and is strongly exposure-time-dependent. A summary of the toxicity of five antibiotics with the lowest EC_50_ values towards green algae and cyanobacteria is presented in Figure 1. The selection of antibiotics was arbitrary based on maximum and minimum EC_50_ values as well as the range values. Two tetracyclines—tetracycline (TCN) and oxytetracycline (OTC)—are characterized by low median effective concentration values towards green algae, *Raphidocelis subcapitata*. The representatives of sulfonamides, sulfadiazine (SDZ) and sulfamethoxazole (SMX) exhibited high toxicity towards *Raphidocelis subcapitata* and *Scenedesmus obliquus.* Spiramycin (SPM) and clarithromycin (CLA) were both characterized by excessive toxicity and a small range gap between highest and lowest EC_50_ values towards green algae. The comprehensive summary of the literature data confirmed the substantial vulnerability of cyanobacteria to antibiotics compared to green algae. Ofloxacin (OFX) and ciprofloxacin (CIP), members of the quinolone family, were highly toxic towards *Synechococcus leopoliensis* and *Microcystis aeruginosa* (low µg/L), followed by clindamycin (CLI), clarithromycin (CLA), and streptomycin (STM).

**Figure 2:**
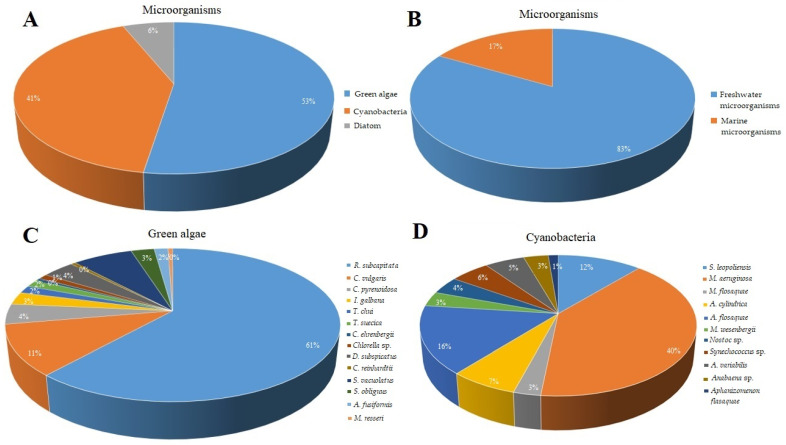
The percent distribution of the toxicological studies on antibiotics based on the number of EC_50_ reports according to different algal groups (multiple data obtained in independent studies). **A**—green algae, cyanobacteria, diatom; **B**—freshwater vs. marine microorganisms; **C**—green algae; **D**—cyanobacteria.

The data on 14 representatives of green algae and 11 cyanobacteria were analyzed. The most frequently applied green algae is freshwater *Raphidocelis subcapitata* with 115 records (out of 189), followed by *Chlorella vulgaris* and *Scenedesmus vacuolatus* with only 22 and 11 records, respectively. Hence, the data on only one species of green algae exceed 60% of the sum of information on the toxicity of antibiotics. The marine species are represented by *Isochrysis galbana*, *Tetraselmis chui,* and *Tetraselmis suecica* with a total of only 11 records (less than 6%). Similarly, the information on cyanobacteria *Microcystis aeruginosa* constitutes 40% of records (57 out of 141), followed by *Anabaena flosaquae* (16%) and the marine microorganism *Synechococcus leopoliensis* (11%) (Figure 2). The data on diatoms are very limited, with only 22 records (6%) on seven different representatives of marine organisms.

### 2.2. Biochemical Endpoints Assessment

The toxicity of antibiotics to green algae, cyanobacteria, and diatom is not limited to growth inhibition. Low, sublethal concentrations of antibiotics (ng/L) lead to growth, photosynthesis, and microcystin synthesis stimulation, which could result in the promotion of cyanobacterial blooms and further affect aquatic ecosystems [24,34]. Liu et al. (2016) [35] reported the hormesis effects in *Microcystin aeruginosa* in response to a 30-day exposure to an environmentally relevant concentration of amoxicillin (AMX). Endpoints defined as independent variables in toxicity tests involving photosynthetic apparatus and an antioxidant system (ex. reactive oxygen species formation, photosynthesis yield, and pigment ratios) are commonly applied in xenobiotic toxicity assessments. The adverse effects of erythromycin (ERY) and sulfamethoxazole (SMX) resulted in membrane integrity damage, reactive oxygen species (ROS) overproduction and consequently increased release of microcystins [36]. In another study, low concentrations (0.001–0.1 µg/L) of ERY led to growth and photosynthetic activity stimulation, while higher levels (40 µg/L) resulted in severe oxidative stress and growth inhibition [37]. The information on the interactions between antibiotics and cyanobacteria is very limited. Some authors focused on gene expression and proteomic responses to interpret the mechanism of antibiotic-induced alterations [38,39]. Sulfamethoxazole (SMX) and ciprofloxacin (CIP) were found to enhance the cell division regulating and transcription-related proteins. The suggested safe threshold for the mixture of both antibiotics was below 10 ng/L (5 ng/L of each antibiotic), which is far lower than reported environmental concentrations [24,35].

Photosynthesis is a fundamental biochemical process based on the conversion of light energy into chemical energy. Photosynthetic organisms possess several protein complexes associated with chloroplasts and thylakoid membranes essential for the light reactions—e.g., photosystems I and II (PSI, PSII) and cytochrome b_6_f [40]. Presumably, the most sensitive and primary site of inhibition caused by environmental pollution is photosystem II—an enzyme complex located in the thylakoid membrane in cyanobacteria and algae [41]. Many studies have demonstrated the reduced photosynthetic efficiency and pigment content as a result of antibiotic exposure [42,43]. Previous research showed that some antibiotics inhibit chlorophyll synthesis by interfering with gene expression of the chloroplast, thus disrupting the physiological process of photochemical reactions [44]. The variations in chlorophyll *a* fluorescence kinetics defined as O, J, I, and P steps of redox states of photosystems PSI and PSII are directly related to the efficiency of electron transfer. Based on the O-J-I-P transient data, biophysical parameters, e.g., F_V_/F_M_ (maximum quantum efficiency of photosystem II), can be calculated [45]. For example, erythromycin was found to adversely affect electron transport and activity of both photosystems (PSI and PSII) in *Microcystis aeruginosa* [46]. A study of the effects of amoxicillin on cyanobacteria *Synechocystis* sp. showed its inhibitory impact on PSII [47]. Some studies reported the adverse effects of antibiotics on the O_2_ evolution processes [29,47,48,49] and chlorophyll, phycobiliproteins (PBPs), and carotenoid contents [29,36,50].

The subjection of autotrophic organisms to stresses such as heat, high-light shock, nutrient deprivation, and xenobiotics pollution can lead to an imbalance between the ability of an organism to inactivate reactive forms of oxygen or repair the potentially related damage and the generation of oxygen intermediates—a phenomenon known as oxidative stress. It leads to the production of reactive oxygen species (ROS), e.g., peroxides and free radicals, levels of which indicate the cellular stress response and are controlled by nonenzymatic antioxidants produced during the de-epoxidation part of the so-called xanthophyll process. The xanthophyll cycle plays a vital role in the photoprotective mechanism against absorption of excess light in photoautotrophic organisms and the formation of ROS, which could lead to the degradation of photosynthetic apparatus and cell death. The epoxidized xanthophylls transform into de-epoxidized forms under the influence of high-intensity light and back into the epoxidized ones under low light, thus minimalizing the amount of energy reaching the photosynthetic reaction centers [23,51]. The monitoring of the xanthophyll cycle enables the detailed observation of physiological responses of microalgae to the oxidative stress induced by the presence of antibiotics in the surrounding medium. Reactive oxygen species removal depends on the activity of enzymatic antioxidants including superoxide dismutase (SOD), catalase (CAT), and glutathione-specific peroxidase (GPX), glutathione-S-transferase (GST) as well as nonenzymatic antioxidants (ascorbate AsA and glutathione GSH) [23]. Glutathione (GSH) as a part of the cellular antioxidant network plays an important role in modulating the response to oxidative stress. It is a redox-active cellular multifunctional molecule part of the essential metabolic pathways involved in detoxification of xenobiotics itself as well as reducing the toxicity of ROS formed as a result of the xenobiotic activity [52]. GSH has been found to play a crucial role in the protection of photosystem I (PSI) from oxidative damage [53]. Another important biomarker of lipid peroxidation and free radical damage to lipids reflecting cellular damage is malondialdehyde (MDA) content. Some studies focused on the link between antibiotic exposure and oxidative stress [38,53,54]. Wang et al. (2017) [55] investigated the effects of thiamphenicol (THI) and florfenicol (FLO) on the antioxidant system and photosynthetic apparatus of *Microcystis flosaquae*. At higher concentrations (>1 µg/L), the levels of SOD and CAT, as well as malondialdehyde (MDA), increased indicating oxidative stress, while at a concentration range of 0.001–1 µg/L, the activities of the antioxidants changed only slightly. Seoane et al. (2014) [56] studied the impact of oxytetracycline (OTC), chloramphenicol (CAP), and florfenicol (FLO) by applying a fluorescein diacetate (FDA) assay to assess the metabolic activity of marine microalgae *Tetraselmis suecica.* The application of other physiological endpoints provided the early detection of significant alteration in cellular content and chlorophyll *a* fluorescence.

The summary of available information on the relationship between antibiotics and early markers of their toxicity is presented in Table S2**.** There is a total of 120 records on 31 different antibiotics belonging to nine classes. The most studied green algae are freshwater *R. subcapitata* and *C. vulgaris* with 16 and 13 records (out of 58), respectively. *T. suecica, Picochlorum oklahomensis,* and *Dunaliella* sp. with a total of only seven records (12%) are the only examples of marine representatives out of a sum of 12 microalgae species. Cyanobacteria are represented by seven different species with *M. aeruginosa* as the most studied microorganism (35 out of 56 records and 63%). The marine species *Chrysosporum ovalisporum* and *Nodularia spumigena* only have nine records (17%). There are only six reports regarding marine species of diatom *Skeletonema costatum*, *Navicula pelliculosa,* and *Phaeodactylum tricornutum* (5% out of the total number of records).

A comprehensive review of the effective concentrations and comparison of different endpoints including pigments contents, oxidative stress, photosynthetic activity, and other biochemical parameters suggests they have a higher sensitivity compared to classical biomass inhibition expressed as growth inhibition or cell density. The alterations of these parameters occurred at a molecular level and lower antibiotic concentrations; therefore, they can be considered as more reliable indicators of the physiological status of affected microorganisms [56,57,58].

## 3. The Mixture Effects

Autotrophic microorganisms are continuously exposed to complex mixtures of substances, including antibiotics. Therefore, it is vital to evaluate the potential interactions between the components of the mixture that could lead to a more significant outcome compared to the impact of substances acting individually [17,59,60]. The risk associated with the presence of mixtures of contaminants may be significantly underestimated if we focus only on individual antibiotics. Previous research showed the apparent synergistic effect of binary mixtures of ciprofloxacin and other antibiotics on the growth of microalgae *Raphidocelis subcapitata* [61]. The same result was observed in the case of the combined toxicity of oxytetracycline, chlortetracycline, and enrofloxacin towards *Ankistrodesmus fusiformis* [62]. The combined effects of ciprofloxacin, tylosin, and lincomycin on two marine diatoms, *Navicula ramosissima* and *Cylindrotheca closterium*, were additive and synergistic, respectively [63]. In the study of the toxicity of a mixture of sulfonamides, including sulfamethoxazole and their transformation products to the microalgae *Scenedesmus vacuolatus*, simple additive effects were reported [64]. Another recent study demonstrated that a mixture of environmentally relevant concentrations of sulfamethoxazole and trimethoprim significantly reduced the growth of three marine microalgae species—*Nannochloropsis oculata*, *Chaetoceros neogracile*, and *Isochrysis galbana*—compared to the effects of individual compounds [28]. Yang et al. (2008) [65] analyzed the growth-inhibiting effects of binary mixtures of 12 different antibacterial compounds towards *Raphidocelis subcapitata*. Potential additive effects were observed in the binary mixtures of sulfonamides, while the combinations of fluoroquinolones, tetracyclines, and macrolides resulted in synergism. The antagonistic effects were found only in the mixtures of triclocarban and norfloxacin, tylosin and norfloxacin, and triclosan.

The presence of low, environmentally relevant concentrations (ng/L) of mixed antibiotics promoted the growth and photosynthesis rate, gene expression, and microcystin synthesis ability of cyanobacteria [26,34,39,66]. Similar effects were observed in the case of binary mixtures of low concentrations of spiramycin and ampicillin, resulting in intracellular microcystin synthesis and release stimulation [25]. A mixture of ciprofloxacin and sulfamethoxazole present at a concentration under the toxicity threshold led to similar results, followed by strong proteomic responses and increased photosynthetic activity [24,67]. Microcystin production, photosynthesis, and growth stimulation could lead to the increased threat of cyanobacteria to the aquatic environment. Moreover, as phytoplankton species are known to exhibit varying sensitivity to the xenobiotics, alterations of the biota composition and species succession are likely.

The toxicological interactions are influenced by the mode of action and pharmacokinetics of individual compounds. The biological sensitivity of the nontarget microorganisms also plays an important role [17,68]. Additionally, the nature of the combined toxicity can be both influenced by the exposure dosage as well as the mixture ratio. The interactions between antibiotics in the mixture varied from antagonistic to synergistic toxicity at a reversed ratio [25,66]. The antagonistic effects can be explained as the suppression of the adverse effects due to similar binding sites resulting in competition. Similarly, the enhancement of the toxicant activity can be attributed to the secondary action of the other component of the mixture or the combined activity at the identical target site. The mechanisms responsible for the variations are not well understood and need further investigation.

The information on interactions between antibiotics and other environmental contaminants is limited. Zhang et al. (2012) [69] investigated the toxicological significance of the complexation of ciprofloxacin and oxytetracycline with three heavy metals (zinc, copper, and cadmium). The toxicity of the antibiotics was altered by the presence of copper and copper sulfate (CuSO4), resulting in an increased growth rate, F_v_/F_m_ value, chlorophyll *a* content, and microcystin synthesis [70]. The synergistic interactions were observed in the mixtures of four antibiotics and glyphosate [67]. A summary of available toxicity data on mixtures of antibiotics including other pharmaceuticals and contaminants is presented in Table 1.

## 4. Environmental Factors Influencing the Toxicity of Antibiotics

The environmental factors limiting the algal growth include the availability of the main nutrients including total phosphorus and nitrogen, trace elements, and light intensity. The same features can impact the sensitivity of microorganisms to environmental pollutants as well as the fate and transport of antibiotics [82]. The biological and physicochemical properties of antibiotics and their transformation products depend on the abiotic properties of the surrounding environment. However, studies focusing on the environmental factors influencing the toxicity of antibiotics are scarce. Nitrogen was found to alter protein synthesis and affect the toxicity of spiramycin to *Microcystis aeruginosa* [83]. Light irradiation causes photodegradation and the formation of potentially toxic by-products. The degradation products of chlortetracycline exhibited higher toxicity towards *S. obliquus* than the parent compound [84]. The pH conditions affect the absorption, light reactivity and the octanol-water partition coefficient (Log k_ow_) and neutral, anionic, cation, and zwitterionic behaviors of the substances, potentially leading to changes in antibiotic activity and toxicity [31]. The presence of the natural organic matter interacting with organic contaminants through adsorption and bonding can change the ecological effects, behavior, and distribution of antibiotics [47]. Borecka et al. (2016) [85] investigated the influence of salinity variations and corresponding toxicity alterations of three sulphonamides (sulfapyridine, sulfamethoxazole, sulfadimethoxine) and trimethoprim towards freshwater green algae *C. vulgaris*. The toxicity of the tested compounds decreased with the increasing salinity probably due to the reduction in the permeability of the algal cell wall and bioavailability of the substances. Rico et al. (2018) [58] studied the effects of temperature, genetic variation, and species competition on the susceptivity of freshwater cyanobacteria *M. aeruginosa* and green algae *S. obliquus* to enrofloxacin. The strain genetic variation and temperature had a limited effect on the algal response, while the results of the competition experiment suggested that environmentally relevant antibiotic concentrations could affect the structure of the phytoplankton communities.

The information on the toxicity of antibiotics under varying multiple environmental conditions is very limited. Moreover, the main focus has been directed towards understanding the ecotoxicological impact of antibiotics on freshwater compartments with less attention being paid to coastal and marine environments [10]. The current data on these compounds referring to freshwater may not apply to seawater as environmental fate, bioavailability, and mode of action differ between freshwater and marine compartments, mainly due to different physicochemical parameters such as pH, salinity, and organic matter composition.

## 5. Conclusions and Future Research Trends

The comprehensive evaluation of the toxicity of antibiotics towards microalgae is currently not possible due to a lack of sufficient data. The current review presented a number of studies on the toxicity of individual and mixtures of antibiotics under different conditions. Significant knowledge gaps were identified, including the underrepresentation of marine species and a focus on just a few representatives of freshwater green algae and cyanobacteria. The information on the toxicity of antibiotics was mainly based on freshwater green algae *R. subcapitata* and cyanobacterium *M. aeruginosa*, while the information on the marine species equaled less than 6% and 11% of the total number of records on green algae and cyanobacteria, respectively. Additionally, the total number of applied species was low with only 14, 11, and 7 for green algae, cyanobacteria, and diatom. In the case of studies focusing on the relationship between antibiotics and early markers of their toxicity apart from biomass endpoints, the information on diatoms constitutes only 5% of the total number of reports, with the main focus on just two freshwater green algae, *R. subcapitata* and *C. vulgaris*, and cyanobacterium *M. aeruginosa* (over 60% of reports). Therefore, there is a need for the application of a wider range of microalgal species with a particular focus on marine microorganisms. The microbial community-based tests apart from the single-species test should be applied to evaluate the disturbance to important ecosystem services and structure composition. Additionally, most of the studies focused on acute effects with standard exposure time (≥96), and only 9% of reports represented the endpoint values based on the exposure time longer than 7 days. The chronic exposure time strongly affects the responses of varied microalgal species, with a significant reduction in the effective concentration. Still, the mechanisms of antibiotic toxicity and algal responses are not well understood. The assessment of the adverse effects of antibiotics on the growth and functioning of microorganisms with the use of different physiological endpoints, including processes involved in the photosynthesis and photoprotective antioxidant mechanisms, will possibly reflect in the greater extent the real environmental impact on the primary producers in fresh, marine and estuarial waters. Insights into biological mechanisms involved in microalgal toxic responses and knowledge about adverse effects that can lead to population shifts of photoautotrophic microorganisms affecting the balance of the aqueous ecosystems are necessary to obtain accurate risk assessments.

In addition, there is little information on the mixture effects and combined environmental factors influencing toxicity. The ecotoxicity of the antibiotic mixture is commonly higher compared to the effects of its single components and could result in considerable toxic effects even at environmentally relevant concentrations. The holistic mixture approach taking into consideration other variable environmental factors such as pH, salinity, organic carbon concentration, and temperature is vital to avoid the underestimation of the actual impact of antibiotics on the ecosystems. Moreover, the reports on the toxicity of the antibiotic transformation products and metabolites are scarce. Some studies showed their higher potency compared to the parent compound.

Complete ecotoxicological information incorporating knowledge on the chronic effects of a wider range of marine species as well as the ecological interactions, underlying modes of action, and mixture effects is crucial to develop adequate risk assessments. Standard toxicity tests offer quick and easy results but are not reliable in terms of reflecting real environmental conditions. Since the marine and the estuarial environment is under intense anthropogenic pressure, studies concerning the impact of emerging contaminants on marine communities are of high importance to predict the changes in ecosystem dynamics and avoid its degradation.

## Figures and Tables

**Table 1:** The summary of available toxicity data on mixtures of antibiotics including other pharmaceuticals and contaminants. The marine and brackish microorganisms are marked in green. (AMP—ampicillin, AMX—amoxicillin, CEF—cefradine, CIP—ciprofloxacin, CLA—clarithromycin, CEP—cephalothin, CPX—cephalexin, CTC—chlortetracycline, DOX—doxycycline, ENR—enrofloxacin, ERY—erythromycin, FLO—florfenicol, FLU—flumequine, GEN—gentamicin, KAN—kanamycin, LCM—lincomycin, LVX—levofloxacin, NOR—norfloxacin, OTC—oxytetracycline, OFX—ofloxacin, OXO—oxolinic acid, PAR—paromomycin sulfate, ROX—roxithromycin, SMX—sulfamethoxazole, SMZ—sulfamethazine, SPM—spiramycin, TCN—tetracycline, TMP—trimethoprim, TOB—tobramycin, TYL—tylosin, VAN—vancomycin, and 7-ACA-7—aminocephalosporanic acid (degradation product of cephalexin and cefradine).

Compounds	Microorganism	Species	Exposure Time (Days)	Endpoint	Interaction	Effective Concentration [mg/L]	Reference
Binary mixtures of AMX, ERY, LVX, NOR, TCN	Green algae	*Raphidocelis* *subcapitata*	3	Growth rate	Synergism	0.01–1500	[17]
	Cyanobacteria	*Anabaena* CPB4337					
CLA and 9 APIs	Green algae	*Raphidocelis* *subcapitata*	3	Growth rate	Buffering effects (synergistic and antagonistic effects)	6.25–100	[71]
Binary mixtures of AMP, AMX, CEP, CIP, GEN, VAN	Green algae	*Raphidocelis* *subcapitata*	3	Growth rate	Synergism	1–50	[61]
SMX and TMP	Green algae	*Isochrysis galbana,* *Chaetoceros* *Neogracile,* *Nannochloropsis oculata*	20	Growth rate	No interactive effect	0.0000075 (SMX) 0.0000085 (TMP)	[28]
SMZ and SMX	Green algae	*Scenedesmus obliquus*	4 (12)	Growth rate, chlorophyll, and carotenoid content, carbohydrate, fatty acid methyl ester (FAMEs)	-	>0.001^1^ (NOEC) 0.15–0.5^2^ 0.5^3^	[72]
Binary mixtures of TMP, SMX, SMZ, CTC, TCN, CIP, NOR, TYL, ROX, CLA	Green algae	*Raphidocelis* *subcapitata*	3	Growth rate	Additive, synergistic and antagonistic effects	0.000001–0.01	[65]
Binary mixtures of SPM, AMP	Cyanobacteria	*Microcystis* *aeruginosa*	7	Growth rate, microcystin synthesis, chlorophyll a content	Synergism and antagonism (mixture ratio-dependent)	>0.001	[25]
OTC, OXO, ERY, FLO, FLU	Green algae	*Raphidocelis* *subcapitata*	2	Chlorophyll fluorescence kinetics	Synergistic and antagonistic effects	0.13–42	[68]
SPM, AMP	Cyanobacteria	*Microcystis* *aeruginosa*	28	Microcystin synthesis, SOD, CAD activity, MDA content, gene expression	Synergism	0.0003	[26]
Binary mixtures of TYL, LCM, CIP	Diatoms	*Cylindrotheca closterium,* *Navicula* *ramosissima*	5	Growth rate	Synergistic and additive effects	1	[63]
AMX, SPM	Cyanobacteria	*Microcystis* *aeruginosa*	7	Growth rate, chlorophyll *a* content, gene expression, microcystin synthesis	Antagonism and synergism at different mix ratios	0.008	[66]
CTC, OTC, ENR	Green algae	*Raphidocelis* *Subcapitata,* *Ankistrodesmus* *fusiformis*	4	Growth rate	Additive, synergistic and antagonistic effects	0.1–10	[62]
AMX, CIP, SPM, SMX, TCN	Cyanobacteria	*Microcystis* *aeruginosa*	14	Growth rate, chlorophyll fluorescence kinetics (Fv/Fm), proteomic responses, gene expression, ROS activity	-	0.00005–0.0005	[39]
Binary mixtures of CIP and 3 APIs	Green algae	*Chlorella* *vulgaris*	4	Growth rate	Synergism	<100	[73]
A mixture of CIP, LCM, OFX, SMX, and 9 APIs	Green algae	*Raphidocelis* *subcapitata*	3	Genotoxic and proteomic effects, chlorophyll and carotenoids content, growth rate	-	0.000026–0.000249	[74]
TMP and 7 APIs	Diatom	*Phaeodactylum* *tricornutum*	3	Growth rate	-	2.4 (EC_10_)	[75]
CIP, SMX	Cyanobacteria	*Microcystis* *aeruginosa*		Growth rate, microcystin synthesis, proteomic responses	Synergism	0.00002–0.0001	[24]
Binary mixtures of CEF, CPX, 7—ACA, TCN, CTC	Green algae	*Raphidocelis* *subcapitata*	3	Growth rate	Additive and antagonistic effects	0.0001–1	[76]
TCN and titanium dioxide nanoparticles (TiO_2_ NPs)	Green algae	*Scenedesmus obliquus*	3	Growth rate	Additive and antagonistic effects	0.15–0.6 (TCN) 1.4–6 (TiO2 NPs)	[77]
DOX/ microplastics	Green algae	*Tetraselmis chuii*	4	Growth rate, chlorophyll *a* content	Synergism	EC_50_ = 22^1^ EC_50_ = 14^2^ In the mixture: EC_50_ = 11^1^ EC_50_ = 7^2^	[78]
CIP, OTC, and 3 metals	Green algae	*Scenedesmus obliquus*	4	Growth rate	Synergistic effects	0.16 (CIP) 0.23 (OTC)	[69]
AMX, SMX, CIP, TCN, and glyphosate	Cyanobacteria	*Microcystis* *aeruginosa*	10	Proteomic responses, chlorophyll fluorescence kinetics (Fv/Fm), microcystin synthesis	Synergism	0.00004–0.0002	[67]
AMX, CIP, SMX, TCN, and copper sulfate (CuSO4)	Cyanobacteria	*Microcystis* *aeruginosa*	20	Growth rate, chlorophyll fluorescence kinetics (Fv/Fm) chlorophyll *a* content and microcystin synthesis, gene expression	Alleviated toxicity of CuSO4 in the presence of antibiotics, increased growth rate, Fv/Fm value, chlorophyll *a* content and microcystin synthesis	0.01–0.05	[34]
KAN, PAR, TOB, and Cu	Green algae	*Chlorella* *pyrenoidosa*			Synergism (weak antagonism)		[79]
CTC and copper (II)	Green algae	*Chlorella* *pyrenoidosa*	4	Chlorophyll fluorescence kinetics (Fv/Fm), MDA, protein content, SOD activity,	Synergism	6.89–37.7	[80]
	Cyanobacteria	*Microcystis* *aeruginosa*					
ENR and cerium oxide nanoparticles (CeO2 NPs	Green algae	*Chlamydomonas reinhardtii*	3	Growth rate, chlorophyll fluorescence kinetics, ROS activity	ENR toxicity decrease	0.01 and 0.1 (ERY) 0.1 (CeO2 NPs)	[81]
	Diatom	*Phaeodactylum* *tricornutum*					

^1^*—*growth inhibition, ^2^*—*pigments content, and ^3^*—*fatty acids composition.

## Data Availability

All data used in this research work are available in the text of the manuscript and supplementary materials.

## Supplementary Materials

The following are available online at https://www.mdpi.com/2223-7747/10/3/591/s1, Table S1. The summary of available toxicity data on individual antibiotics including EC_50_ values based on growth suppression, photosynthetic yield, and oxidative stress markers. Table S2. The summary of available toxicity data on individual antibiotics regarding various toxicity markers.
