# Supplementary material for: The Toxic Effects of Antibiotics on Freshwater and Marine Photosynthetic Microorganisms: State of the Art
Source: Plants (Basel). 2021 Mar 21;10(3):591. doi: 10.3390/plants10030591 (PMC8004086; doi:10.3390/plants10030591)
Supplement: Supplementary file 1 [file plants-10-00591-s001.pdf]

plants

Table S1. The summary of available ecotoxicity data on individual antibiotics including EC_{50} values based on growth suppression, photosynthetic yield, and oxidative stress markers. The marine and brackish microorganisms are marked in green. (ATC- anhydrotetracycline (tetracycline degradation product), AMP- ampicillin, AMX- amoxicillin, AZM- azithromycin, CAP- chloramphenicol, CFZ- cefazolin, CIP- ciprofloxacin, CLA- clarithromycin, CEP-cephalotin, CLF- clinafloxacin, CLI- clindamycin, CTC – chlortetracycline, CTX- cefotaxime, DOX– doxycycline, DSM- Dihydrostreptomycin, ENR- enrofloxacin, ERY – erythromycin, ETC- pitetracycline (tetracycline degradation product), FLO- florfenicol, FLU- flumequine, GEN- gentamicin, GFX- gatifloxacin, LCM- lincomycin, LMX- lomefloxacin, LVX- levofloxacin, , LZD- linezolid, MEC- mecillinam, MIN – minocycline, MTZ- metronizadole, MXF- moxifloxacin , NOR- norfloxacin, OLX- olaquindox, OTC- oxytetracycline, OFX- ofloxacin, OXO- oxolinic acid, PEN- penicillin, , ROX- roxithomycin, RIF- rifampicin, SAT- sulfacetamide, SCP- sulfachlorpyridazine, SDM- sulfadimidine, SDX sulfadimethoxine, SDZ- sulfadiazine, SFX- sarafloxacin, SLM- sufamerazine, SLP- sulfapyridine, SLT – sulfathiazole, SLX- sulfisoxazole, SMM- sulfamonomethoxine, SMP- sulfamethoxypyridazine, SMT- sulfamethiazole, SMX- sulfamethoxazole, SMZ- sulfamethazine, SPM- spiramycin, STM- streptomycin, SUL- Sulfaguanidine, TCN- tetracycline, TIA – tiamulin, TGC- tigecycline, THI- thiamphenicol, TMP- trimethoprim, TYL- tylosin, QUI- quinocetone, VAN-vancomycin, 7-ACA - 7-aminocephalosporanic acid (degradation product of cephalexin and cefradine)

Antibiotic group	Substance	Microorganism	Species	Exposure time (days)	EC_{50} [mg/L]	Reference
Aminoglycosides	DSM	Green algae	*Raphidocelis subcapitata*	3	0.107	1
	GEN	Green algae	*Raphidocelis subcapitata*	3	19.2	2
		Cyanobacteria	*Synechococcus leopoliensis*	3	0.069	3
	STM	Green algae	*Chlorella vulgaris*	4	20.08	4
			Raphidocelis subcapitata	1	1.5[1]	5
				3	0.133	6
		Cyanobacteria	*Microcystis aeruginosa*	1	0.034[1]	5
				4	0.29	4
				7	0.007	6
Amphenicols	CAP	Green algae	*Chlorella pyrenoidosa*	4	14	7
			Raphidocelis subcapitata	3	137	8
			Isochrysis galbana	4	41	7
			Tetraselmis chui	4	4	7
			Tetraselmis suecica	4	11.16	9
		Diatom	*Chaetoceros gracilis*	15	>0.3	10
	FLO	Green algae	*Raphidocelis subcapitata*	4	5.02	11
				2	2.3-3.7[1]	12
			Chlorella pyrenoidosa	4	215	7
			Isochrysis galbana	4	8	7
			Tetraselmis chui	4	1.3	7
			Tetraselmis suecica	4	9.03	9
		Cyanobacteria	*Microcystis flosaquae*	7	≈ 0.05	13
		Diatom	*Skeletonema costatum*	4	5.043	14
	THI	Green algae	*Raphidocelis*	3	8.86	1

			subcapitata			
			Chlorella pyrenoidosa	4	1238	7
			Isochrysis galbana	4	152	7
			Tetraselmis chui	4	38	7
		Cyanobacteria	*Anabaena cylindrica*	6	1.3	15
			Anabaena flosaquae	6	13	15
			Anabaena variabilis	6	14	15
			Microcystis aeruginosa	6	0.32	15
			Microcystis flosaquae	7	≈ 0.01	13
			Microcystis wesenbergii	6	0.43	15
			Nostoc sp. PCC 7120	6	3.5	15
			Synechococcus leopoliensis	6	0.36	15
			Synechococcus sp.	6	0.67	15
Beta-lactams	AMX	Green algae	*Chlorella sp.*	4	853.54±0.27	16
			Closterium ehrenbergii	4	>50	17
			Chlorella pyrenoidosa	6	>2000	18
			Raphidocelis subcapitata	4	>50	17
				3	>2000	2
				3	>1500	19
				4	3.24	11
				3	>250	20
		Cyanobacteria	*Anabaena flosaquae*	3	0.1	21
			Anabaena cylindrica	6	7.66	18
			Anabaena sp. CPB4337	3	56.3	19
			Microcystis aeruginosa	7	0.0037	20
				7	0.008	22
			Synechococcus leopoliensis	4	0.0022	17
	AMP	Green algae	*Raphidocelis subcapitata*	3	>1000	1
				3	>2000	2
		Cyanobacteria	*Anabaena cylindrica*	6	0.14	15
			Anabaena flosaquae	6	3.3	15
			Anabaena variabilis	6	2.2	15
			Microcystis aeruginosa	6	0.0002	15
				4	0.012	4
			Microcystis wesenbergii	6	0.013	15
			Synechococcus leopoliensis	6	0.083	15
			Synechococcus sp.	6	0.069	15
	CEP	Green algae	*Raphidocelis subcapitata*	3	>600	2
	CTX	Green algae	*Raphidocelis subcapitata*	4	3.15	11
	MEC	Cyanobacteria	*Microcystis aeruginosa*	3	0.06	95
	PEN	Cyanobacteria	*Microcystis aeruginosa*	7	0.006	6

Cephalo-sporin	CFZ	Green algae	*Raphidocelis subcapitata*	3	>1000	1
	7-ACA*	Green algae	*Raphidocelis subcapitata*	3	0.775	23
Diaminopyrimidine	TMP	Green algae	*Chlorella vulgaris*	3	90.86	24
				4	>100	25
			Desmodesmus subspicatus	4	>79	25
			Raphidocelis subcapitata	4	83.8	26
				3	129	27
				3	80.3	1
				4	>89.1	25
				1	>9[1]	5
				3	0.104	28
				3	130	20
				3	110	95
		Cyanobacteria	*Anabaena flosaquae*	3	253	27
				4	>99	25
				6	>200	15
				3	183.7	21
			Anabaena variabilis	6	11	15
			Microcystis aeruginosa	7	112	20
				6	150	15
				1	6.9[1]	5
				3	112	95
			Nostoc sp. PCC 7120	6	53	15
			Synechococcus leopoliensis	3	182	21
				4	>100	25
		Diatom	*Navicula pelliculosa*	4	20.4<br>39.5[1]	25
			Phaeodactylum tricornutum	4	21.6	25
				3	2.4	29
Glyco-peptide	VAN	Green algae	*Raphidocelis subcapitata*	3	724	2
Lincosamides	CLI	Cyanobacteria	*Anabaena flosaquae*	3	0.0296	21
			Raphidocelis subcapitata	3	0.01	30
	LCM	Green algae	*Chlorella vulgaris*	4	>91.7	25
			Desmodesmus subspicatus	4	>76.7<br>32.2[1]	25
			Raphidocelis subcapitata	4	9.8<br>4.8[1]	25
				4	1.51	17
				3	0.07	31
		Cyanobacteria	*Anabaena flosaquae*	4	0.487<br>1.93[1]	25

				4	0.195	17
			Synechococcus leopoliensis	4	0.038	25
		Diatom	*Cylindrotheca closterium*	5	14.16	32
			Navicula ramosissima	5	11.8	32
			Navicula pelliculosa	4	>62.5	25
			Cyclotella meneghiniana	4	1.63	17
			Phaeodactylum tricornutum	4	>91.7	25
Macrolides	AZM	Green algae	*Chlorella sp.*	4	0.33±0.05	16
			Raphidocelis subcapitata	4	8.07	11
				4	0.019	33
	CLA	Green algae	*Chlorella sp.*	4	0.59±0.004	16
			Desmodesmus subspicatus	3	0.0371	34
			Raphidocelis subcapitata	3	0.046	28
				3	0.23	30
				3	0.0069	35
				4	0.011	36
				4	0.012	33
				3	0.002	31
		Cyanobacteria	*Anabaena flosaquae*	3	0.0121	34
				3	0.0132	21
			Synechococcus leopoliensis	3	0.0152	21
	ERY	Green algae	*Chlorella vulgaris*	3	33.8	1
				4	85.7	37
			Chlamydomonas reinhardtii	3	0.36<br>0.59[1]<br><0.1[2]	38
			Raphidocelis subcapitata	3	0.35	19
				3	0.02	31
				3	0.0366	1
				2	0.13-0.24[1]	12
		Cyanobacteria	*Anabaena cylindrica*	6	0.035	15
			Anabaena sp. CPB4337	3	0.022	19
			Anabaena flosaquae	3	0.3449	21
				6	0.27	15
			Anabaena variabilis NIES-23	6	0.43	15
			Microcystis aeruginosa	6	0.023	15
				4	0.023	39
			Microcystis wesenbergii	6	0.023	15

			Nostoc sp. PCC 7120	6	0.02	15
			Synechococcus leopoliensis	6	0.16	15
			Synechococcus sp.	6	0.23	15
				5	0.1	43
		Diatom	*Phaeodactylum tricornutum*	3	1.31<br>8.26[1]<br><0.1[2]	38
			Chaetoceros gracilis	15	>0.5	10
	ROX	Green algae	*Chlorella pyrenoidosa*	4	2.87	44
			Raphidocelis subcapitata	3	0.047	28
	SPM	Green algae	*Chlorella pyrenoidosa*	6	4.58	18
			Raphidocelis subcapitata	3	2.3	6
		Cyanobacteria	*Anabaena cylindrica*	6	0.0384	18
			Microcystis aeruginosa	7	0.005	6
				7	0.00115	22
				7	0.00114	13
	TYL	Green algae	*Chlorella vulgaris*	4	>74.3	25
			Desmodesmus subspicatus	4	35<br>16[1]	25
			Raphidocelis subcapitata	4	4.3<br>1.9[1]	25
				1	0.0089[1]	5
				3	1.38	6
				3	0.21	28
				3	0.411	1
		Cyanobacteria	*Anabaena flosaquae*	4	0.054<br>0.3[1]	25
			Microcystis aeruginosa	7	0.034	6
				1	0.29	5
			Synechococcus leopoliensis	4	0.083	25
		Diatom	*Cylindrotheca closterium*	5	0.27	32
			Navicula ramosissima	5	0.99	32
			Navicula pelliculosa	4	4.03<br>6.7 [1]	25
			Phaeodactylum tricornutum	4	5.22	25
Nitroimidazoles	MTZ	Green algae	*Chlorella vulgaris*	3	12.5	40
			Raphidocelis subcapitata	4	3.22	11
				3	40.4	40
Oxazolidinones	LZD	Cyanobacteria	*Anabaena flosaquae*	3	2.12	21

Pleu-romu-tilins	TIA	Green algae	*Raphidocelis subcapitata*	3	0.165	6
		Cyanobacteria	*Microcystis aeruginosa*	7	0.003	6
Ri-famycins	RIF	Green algae	*Raphidocelis subcapitata*	4	3.91	11
Sulphonamides	SCP	Green algae	*Scenedesmus vacuolatus*	1	32.25	41
	SAT	Green algae	*Chlorella vulgaris*	2	13.28	47
	SDM	Green algae	*Scenedesmus vacuolatus*	1	19.52	41
	SDZ	Green algae	*Chlorella vulgaris*	2	1.22	47
			Isochrysis galbana	4	1.44	42
			Raphidocelis subcapitata	3	7.8	20
				3	2.19	1
			Scenedesmus vacuolatus	1	2.22	41
		Cyanobacteria	*Microcystis aeruginosa*	7	0.135	20
		Diatom	*Phaeodactylum tricornutum*	4	0.11	42
	SDX	Green algae	*Chlorella vulgaris*	3	11.2	1
				3	7.65	24
			Raphidocelis subcapitata	3	2.3	1
			Scenedesmus vacuolatus	1	9.85	41
		Cyanobacteria	*Anabaena cylindrica*	6	480	15
			Anabaena flosaquae	6	>2000	15
			Anabaena variabilis	6	1500	15
			Microcystis aeruginosa	6	500	15
			Microcystis wesenbergii	6	470	15
			Nostoc sp. PCC 7120	6	>2000	15
			Synechococcus leopoliensis	6	1100	15
			Synechococcus sp.	6	760	15
	SLM	Green algae	*Scenedesmus vacuolatus*	1	11.9	41
	SLP	Green algae	*Scenedesmus vacuolatus*	1	5.28	41
			Chlorella vulgaris	3	1	24
	SLT	Green algae	*Chlorella vulgaris*	2	17.7	47
			Scenedesmus vacuolatus	1	13.1	41
	SLX	Green algae	*Scenedesmus vacuolatus*	1	18.98	41
	SMM	Green algae	*Raphidocelis subcapitata*	4	5.3	11

	SMP	Green algae	*Scenedesmus vacuolatus*	1	3.82	41
	SMT	Green algae	*Scenedesmus vacuolatus*	1	24.94	41
	SMZ	Green algae	*Raphidocelis subcapitata*	3	0.103	28
			Scenedesmus obliquus	4	0.15	46
	SMX	Green algae	*Chlorella vulgaris*	2	1.57	47
				3	1.51	24
			Raphidocelis subcapitata	3	0.52	31
				4	0.146	48
				3	0.103	28
				1	>9[1]	5
				3	1.53	1
			Scenedesmus obliquus	4	0.15	46
			Scenedesmus vacuolatus	1	1.54	41
		Cyanobacteria	*Anabaena flosaquae*	3	73.9	21
			Microcystis aeruginosa	1	0.55	5
			Synechococcus leopoliensis	4	0.0268	48
				3	1.18	21
		Diatom	*Cyclotella meneghiniana*	4	0.0024	48
	SUL	Green algae	*Scenedesmus vacuolatus*	1	3.42	41
Tetracyclines	ATC*	Green algae	*Chlorella vulgaris*	4	5.96	49
	CTC	Green algae	*Ankistrodesmus fusiformis*	4	3.23 ± 0.53	50
			Chlorella pyrenoidosa	4	32.6	51
			Raphidocelis subcapitata	4	1.19 ± 0.53	50
				3	3.1	6
				3	0.103	28
				3	0.0047	23
		Cyanobacteria	*Microcystis aeruginosa*	7	0.05	6
				9	0.01-1	52
				4	13.1	51
	DOX	Green algae	*Raphidocelis subcapitata*	4	5.92	11
	ETC*	Green algae	*Chlorella vulgaris*	4	8.42	49
	MIN	Cyanobacteria	*Microcystis aeruginosa*	12	0.421	53
	OTC	Green algae	*Ankistrodesmus fusiformis*	4	7.15	54
				4	4.17 ± 3.79	50
			Chlorella vulgaris	2	6.4	55
				3	7.05	1
			Isochrysis galbana	4	6.43	42

			Raphidocelis subcapitata	4	3.1	56
				3	0.17	31
				2	0.47-2.0[1]	12
				4	0.92 ± 0.30	54
				3	1.04	27
				4	0.64 ± 0.38	50
				4	5.07	11
				1	0.6[1]	5
				3	0.342	1
				3	4.5	20
			Tetraselmis suecica	4	17.25	9
		Cyanobacteria	*Anabaena cylindrica*	6	0.032	15
			Aphanizomenon flosaquae	3	2.7	27
				6	0.39	15
			Anabaena variabilis	6	0.36	15
			Microcystis aeruginosa	7	0.207	20
				6	0.23	15
				7	0.09	6
				1	5.4[1]	5
				9	0.01-1	52
			Microcystis wesenbergii	6	0.35	15
			Nostoc sp. PCC 7120	6	7.0	15
			Synechococcus leopoliensis	6	1.1	15
			Synechococcus sp.	6	2.0	15
		Diatom	*Phaeodactylum tricornutum*	4	1.73	42
	TCN	Green algae	*Chlorella vulgaris*	4	7.73	49
			Raphidocelis subcapitata	3	3.31	19
				3	0.103	28
				7	>1	57
				3	2.2	6
				3	0.005	23
		Cyanobacteria	*Anabaena* sp.CPB4337	3	6.2	19
			Microcystis aeruginosa	3	0.09	6
				7	>1	57
				9	0.01-1	52
	TGC	Green algae	*Chlorella pyrenoidosa*	6	6.2	18
		Cyanobacteria	*Anabaena cylindrica*	6	0.062	18
Quin-olones	CIP	Green algae	*Chlorella vulgaris*	4	29.09	58
				4	20.6	59
			Desmodesmus subspicatus	3	0.008	60
				3	8.8	61
			Raphidocelis subcapitata	3	11.3	2

			5	18.7	62
			4	39	63
			4	4.67	11
			3	0.103	28
			7	2.97	95
	Cyanobacteria	*Anabaena flosaquae*	3	0.0102	60
			3	0.0373	21
		Microcystis aeruginosa		0.01724	64
			3	0.005	95
			5	0.017	62
	Diatom	*Cylindrotheca closterium*	5	55.43	32
		Navicula ramosissima	5	72.12	32
CLF	Green algae	*Raphidocelis subcapitata*	5	1.1	62
	Cyanobacteria	*Microcystis aeruginosa*	5	0.103	62
ENR	Green algae	*Ankistrodesmus fusiformis*	4	10.6 ± 1.28	50
		Chlorella vulgaris	4	32.49	65
			4	124.5	37
		Desmodesmus subspicatus	2	5.57	60
		Micractinium resseri	4	28.85	65
		Raphidocelis subcapitata	5	3.1	62
			4	5.18 ± 3.80	50
		Scenedesmus obliquus	4	57.61	65
			3	38 (20°C)<br>41 (30°C)	66
			4	59.16	67
	Cyanobacteria	*Anabaena flosaquae*	3	0.173	60
		Microcystis aeruginosa	5	0.049	62
		Microcystis aeruginosa SAG 1785	3	0.031	66
		Microcystis aeruginosa PCC7806	3	0.029 (30°C)<br>0.047 (20°C)	66
		Microcystis aeruginosa V131	3	0.029	66
FLU	Green algae	*Raphidocelis subcapitata*	1	16[1]	5
			4	2.6	56
			5	5	62
			3	5.0	20
			2	8.1-9.3[1]	12
	Cyanobacteria	*Microcystis aeruginosa*	5	1.960	62
			7	0.159	20
			1	>8.8[1]	5
GFX	Cyanobacteria	*Microcystis aeruginosa*	4	25.3	68
LVX	Green algae	*Raphidocelis subcapitata*	5	7.4	62
			3	>120	19

				3	1.2	36
		Cyanobacteria	*Anabaena* sp. CPB4337	3	4.8	19
			Microcystis aeruginosa	5	0.0079	62
			Microcystis flosaquae	7	>0.01<0.04[2]	69
	LMX	Green algae	*Raphidocelis subcapitata*	5	22.7	62
		Cyanobacteria	*Microcystis aeruginosa*	5	0.186	62
	MXF	Cyanobacteria	*Microcystis aeruginosa*	4	60.34	68
	NOR	Green algae	*Chlorella vulgaris*	3	10.4	1
			Raphidocelis subcapitata	3	>80	19
				3	16.6	1
				4	3.73	11
				3	0.103	28
			Scenedesmus obliquus	4	50.18	93
		Cyanobacteria	*Anabaena* sp. CPB4337	3	5.6	19
			Anabaena cylindrica	6	0.053	15
			Anabaena flosaquae	6	0.29	15
			Anabaena variabilis	6	0.19	15
			Microcystis aeruginosa	6	0.062	15
			Microcystis wesenbergii	6	0.038	15
			Nostoc sp. PCC 7120	6	1.7	15
			Synechococcus leopoliensis	6	0.63	15
			Synechococcus sp.	6	0.63	15
	OFX	Green algae	*Raphidocelis subcapitata*	3	12.1	62
				4	4.93	11
				3	1.44	31
		Cyanobacteria	*Microcystis aeruginosa*	5	0.021	62
			Synechococcus leopoliensis	4	0.016	48
		Diatom	*Cyclotella meneghiniana*	4	0.09	48
	OXO	Green algae	*Raphidocelis subcapitata*	3	16	20
				2	37[1]	12
		Cyanobacteria	*Microcystis aeruginosa*	7	0.180	20
	SFX	Green algae	*Raphidocelis subcapitata*	3	16	20
		Cyanobacteria	*Microcystis aeruginosa*	7	0.015	20
Quinoxa-lines	OLX	Green algae	*Raphidocelis subcapitata*	3	40	6
		Cyanobacteria	*Microcystis aeruginosa*	7	5.1	6
	QUI	Green algae	*Raphidocelis subcapitata*	4	4.82	11

1- photosynthetic activity, 2 – oxidative stress, * – degradation products

Table 2S. The summary of available ecotoxicity data on individual antibiotics regarding various toxicity markers. The marine and brackish microorganisms are marked in green. (ATC- anhydrotetracycline (tetracycline degradation product), AMP- ampicillin, AMX- amoxicillin, CAP-chloramphenicol, CEF- cefradine, CIP- ciprofloxacin, CLA- clarithromycin, CTC – chlortetracycline, ENR- enrofloxacin, ERY – erythromycin, ETC- pitetracycline (tetracycline degradation product), FLO- florfenicol, , GEN- gentamicin, GFX- gatifloxacin, KAN- kanamycin, LCM- lincomycin, MXF- moxifloxacin , NOR- norfloxacin, OTC- oxytetracycline, OFX- ofloxacin, ROX- roxithomycin, SDZ- sulfadiazine, SMX-sulfamethoxazole, SMZ-sulfamethazine, SPM- spiramycin, STM- streptomycin, TCN- tetracycline, TGC- tigacycline, THI- thiamphenicol, TMP- trimethoprim, TYL-tylosin)

Antibiotic group	Substance	Microorganism	Species	Exposure time (days)	Endpoint	Effective concentration [mg/L]	Reference
Aminoglycosides	GEN	Cyanobacteria	*Synechocystis* sp.	1	GSH precursor and cellular GSH content	0.01[1]	70
	KAN	Green algae	*Dictyosphaerium pulchellum*	11	Chlorophyll fluorescence kinetics, protein content	5[2,9]	71
			Micractinium pusillum	11	Chlorophyll fluorescence kinetics, protein content	5[9]	71
		Green algae	*Chlorella vulgaris*	4	Chlorophyll and phycocyanobilin content, MDA content and electrolyte leakage (membrane damage), SOD, POD, CAT activities, photosynthesis-related gene transcription inhibition	10[1,5]	4
	STM	Green algae	*Dunaliella* sp.	22	Oxygen evolution, chlorophyll content, chlorophyll fluorescence kinetics	>50[2,5]	72
			Picochlorum oklahomensis	22	Oxygen evolution, chlorophyll content, chlorophyll fluorescence kinetics (F_v/F_m)	>50[2,5]	72
		Cyanobacteria	*Microcystis aeruginosa*	4	Chlorophyll and phycocyanobilin content, MDA content and electrolyte leakage (membrane damage), SOD, POD, CAT activities, photosynthesis-related gene transcription inhibition	0.1[1,5]	4

Amphenicols	CAP	Green algae	*Tetraselmis suecica*	4	FDA metabolic activity, chlorophyll fluorescence kinetics, chlorophyll *a* content	2.52[2,5,10]	9
	FLO	Green algae	*Tetraselmis suecica*	4	FDA metabolic activity, chlorophyll fluorescence kinetics, chlorophyll *a* content	2.5[2,5]	9
		Cyanobacteria	*Microcystis flosaquae*	7	CAT, SOD activity, MDA content, chlorophyll a content, chlorophyll fluorescence kinetics (F_v/F_m)	0.000001-0.001[5]<br>>0.001[1,2]	13
		Diatom	*Skeletonema costatum*	4	Chlorophyll *a* content, chlorophyll fluorescence kinetics (F_v/F_m), ROS overproduction, FDA metabolic activity (membrane damage)	>1.0[1]<br>>4.0[2,5]	14
	THI	Cyanobacteria	*Microcystis flosaquae*	7	CAD, SOD activity, MDA content, chlorophyll a content, chlorophyll fluorescence kinetics (F_v/F_m)	0.000001-0.001[5]<br>>0.001[1,2]	13
Beta-lactams	AMX	Green algae	*Chlorella pyrenoidosa*	6	SOD, CAT activity, MDA content, chlorophyll content	200[1]	18
		Cyanobacteria	*Anabaena cylindrica*	6	SOD, CAT activity, MDA content, chlorophyll content	0.015[1]	18
			Microcystis aeruginosa	7	Microcystins synthesis alteration	0.0001 to 0.001[7]	22
				7	SOD, CAD, POD activity	0.0005[1]	73
				30	Photosynthetic activity and microcystins synthesis stimulation, proteomic responses, metabolism of carbohydrate and nucleoside phosphate	0.001-0.0003[2,7]	74
				1	GSH simulation, SOD activity, MDA content, chlorophyll *a* content	20[1]	75
			Synechocystis sp.	0.5 (12h)	Chlorophyll fluorescence kinetics, O_2 evolution	5[2,11]	76

	AMP	Green algae	*Dunaliella* sp.	22	Oxygen evolution, chlorophyll content, chlorophyll fluorescence kinetics (F_v/F_m)	>50[2,5]	72
			Picochlorum oklahomensis	22	Oxygen evolution, chlorophyll content, chlorophyll fluorescence kinetics (F_v/F_m)	>50[2,5]	72
		Cyanobacteria	*Microcystis aeruginosa*	30	Growth stimulation and microcystin production, CAD, SOD activity, MDA content	0.0003[7]	77
				7	Microcystin synthesis, chlorophyll *a* content	0.00025 to 0.0025[7]	45
	CEF	Green algae	*Scenedesmus obliquus*	6	Chlorophyll *a* content	0.5[5]	78
		Cyanobacteria	*Microcystis aeruginosa*	1	GSH simulation, SOD activity, MDA content, chlorophyll *a* content	10[1]	75
				6	Chlorophyll *a* content	0.5[5]	78
Diaminopyrimidine	TMP	Green algae	*Desmodesmus subspicatus*	4	O_2 evolution, carotenoids and chlorophyll *a* content, chlorophyll fluorescence kinetics	16[2]	25
			Raphidocelis subcapitata	4	O_2 evolution, carotenoids and chlorophyll *a* content, chlorophyll fluorescence kinetics	1.9[2]	25
		Cyanobacteria	*Anabaena flosaquae*	4	O_2 evolution, carotenoids and chlorophyll *a* content, chlorophyll fluorescence kinetics	>99[2]	25
		Diatom	*Navicula pelliculosa*	4	O_2 evolution, carotenoids and chlorophyll *a* content, chlorophyll fluorescence kinetics	39.5[2]	25
Lincosamides	LCM	Green algae	*Desmodesmus subspicatus*	4	O_2 evolution, carotenoids and chlorophyll *a* content, chlorophyll fluorescence kinetics	32.2[2]	25
			Raphidocelis subcapitata	4	O_2 evolution, carotenoids and chlorophyll *a* content, chlorophyll fluorescence kinetics	4.8[2]	25

		Cyanobacteria	*Anabaena flosaquae*	4	O_2 evolution, carotenoids and chlorophyll *a* content, chlorophyll fluorescence kinetics	1.93[2]	25
		Diatom	*Navicula pelliculosa*	4	O_2 evolution, carotenoids and chlorophyll *a* content, chlorophyll fluorescence kinetics	>62.5[2]	25
Macrolides	CLA	Green algae	*Chlorella vulgaris*	7	SOD, CAT, GSH, GST, GPX activity, chlorophyll *a*, *b*, and carotenoid contents	0.04[1]<br>0.02[5]	79
			Raphidocelis subcapitata	5	SOD activity and LPO levels, cellular energy allocation (CEA)	0.0056 (LOEC)	81
				7	SOD, CAT, GSH, GST, GPX activity, chlorophyll *a*, *b*, and carotenoid contents	0.02[1,5]	79
	ERY	Green algae	*Chlorella vulgaris*	4	MDA and GS contents, chlorophyll *a* content	0.021[1,5]	77
			Chlamydomonas reinhardtii	3	Chlorophyll fluorescence kinetics, ROS production, the cell membrane integrity	<0.1[1]<br>0.59[2]	38
			Raphidocelis subcapitata	4	CAD, SOD, GPX, GST activity, the ascorbate - glutathione cycle, the xanthophyll cycle	0.3 [1]<br>0.3 [3]<br>0.06 [4]	80
				5	SOD activity and LPO levels, cellular energy allocation (CEA)	0.012 (LOEC)	81
				3	Chlorophyll fluorescence kinetics, chlorophyll a content, hyperpolarization of mitochondrial membrane, ROS activity, GSH content	0.002 [5]<br>0.038[1,2]	82
				4	Chlorophyll *a* content, photosynthetic rate, chlorophyll biosynthesis inhibition	0.3[2]<br>0.18[5]<br>0.06 [6]	83

	Cyanobacteria	*Microcystis aeruginosa*	4	ROS, MDA, SOD content, microcystins synthesis, Chlorophyll *a* and carotenoid content, chlorophyll fluorescence kinetics	0.1510[1] >0.01[2,7] 0.02[5]	94
			7	Cell membrane hyperpolarization, chlorophyll *a* fluorescence, microcystin synthesis	0.0005[2] and 0.005[2] 0.0005[7] to 0.5[7]	92
		Microcystis flosaquae	8	SOD, CAT, MDA activity, chlorophyll a fluorescence (Fv/Fm) , chlorophyll *a* content	0.04[1] 0.001–0.1[2]	85
	Diatom	*Phaeodactylum tricornutum*	3	Chlorophyll fluorescence kinetics, ROS production, the cell membrane integrity	<0.1[1] 8.26[2]	38
ROX	Green algae	*Chlorella pyrenoidosa*	4 (21)	SOD, CAT activity, MDA content, chlorophyll biosynthesis inhibition,	1-2[1,6]	41
SPM	Green algae	*Chlorella pyrenoidosa*	6	SOD, CAT activity, MDA content, chlorophyll content	5[1]	92
	Cyanobacteria	*Anabaena cylindrica*	6	SOD, CAT activity, MDA content, chlorophyll content	0.1[1]	92
		Microcystis aeruginosa	7	Microcystins synthesis alteration	0.0001 to 0.001[7]	22
			7	SOD, CAD, POD activity	0.0005 to 1[1]	73
			7	Microcystin synthesis, chlorophyll *a* content	0.00025 to 0.0025[7]	45
			30	Growth stimulation and microcystin production, oxidative stress (CAD, SOD activity, MDA content)	0.0003[7]	37
TYL	Green algae	*Desmodesmus subspicatus*	4	O_2 evolution, carotenoids and chlorophyll *a* content, chlorophyll fluorescence kinetics	16[2]	25
		Raphidocelis subcapitata	4	O_2 evolution, carotenoids and chlorophyll *a* content, chlorophyll fluorescence kinetics	1.9[2]	25

		Cyanobacteria	*Anabaena flosaquae*	4	O_2 evolution, carotenoids and chlorophyll *a* content, chlorophyll fluorescence kinetics	0.3[2]	25
		Diatom	*Navicula pelliculosa*	4	O_2 evolution, carotenoids and chlorophyll *a* content, chlorophyll fluorescence kinetics	6.7[2]	25
Sulphonamides	SDZ	Green algae	*Chlorella vulgaris*	16	Chlorophyll fluorescence kinetics (F_v/F_m), SOD, CAT, MDA activity, chlorophyll *a* content	5[5]<br>5[1]	86
		Cyanobacteria	*Chrysosporum ovalisporum*	16	Chlorophyll fluorescence kinetics (F_v/F_m), SOD, CAT, MDA activity, chlorophyll a content	1[5] mg/L<br>50[1]	86
				11	Phycobiliproteins content, and alkaline phosphatase (ALP) activity, chlorophyll *a* content	1[5]	92
	SMZ	Green algae	*Chlorella vulgaris*	16	Chlorophyll fluorescence kinetics (F_v/F_m), SOD, CAT, MDA activity, chlorophyll a content	5[5]<br>50[1]	86
			Scenedesmus obliquus	4	The unsaturated fatty acid methyl esters (FAMEs) content, the chlorophyll and carotenoid content	>0.15[5]	46
		Cyanobacteria	*Chrysosporum ovalisporum*	16	Chlorophyll fluorescence kinetics (Fv/Fm), SOD, CAT, MDA activity, chlorophyll a content	20[1]	86
				11	Phycobiliproteins (PBPs) content, and alkaline phosphatase (ALP) activity, chlorophyll *a* content	1[5]	18
	SMX	Green algae	*Raphidocelis subcapitata*	4	Chlorophyll *a* content, photosynthetic rate, chlorophyll biosynthesis inhibition	>1.5[5]<br>0.5[6]	83
				4	CAD, SOD, GPX and GST activity, the ascorbate - glutathione cycle, the xanthophyll cycle	2.5[1]<br><1.5[3]<br>0.5[4]	80

				5	SOD activity and LPO levels, cellular energy allocation (CEA)	0.4[1]	81
			Scenedesmus obliquus	4	The unsaturated fatty acid methyl esters (FAMEs) content, the chlorophyll and carotenoid content	>0.15[5]	46
		Cyanobacteria	*Microcystis aeruginosa*	7	Cell membrane hyperpolarization, chlorophyll a fluorescence, microcystin synthesis	0.05[2,7] and 0.125[2,7]	84
Tetracyclines	ATC*	Green algae	*Chlorella vulgaris*	4	Cell structure, SOD and CAT content	> 5[1]<br>< 2.5 [8]<br>5[1]	49
	CTC	Green algae	*Scenedesmus obliquus*	10	Chlorophyll *a* content	0.5[5]	87
		Cyanobacteria	*Microcystis aeruginosa*	9	The cell density, chlorophyll *a* and protein content, SOD, CAT, POD activities	0.05[5,9]<br>0.01[1]	52
				6	Microcystin synthesis	1.86[7] (EC_{10})	88
				27	Microcystin synthesis	0.1-0.25	89
				10	Chlorophyll *a* content	0.5[5]	87
	ETC*	Green algae	*Chlorella vulgaris*	4	Cell structure, SOD and CAT content	> 5[1]<br>< 2.5[8]<br>10[1]	49
	OTC	Green algae	*Chlorella vulgaris*	11	Chlorophyll fluorescence kinetics (F_v/F_m), chlorophyll *a* and pheophytin content	0.1[5]<br>1[2]	90
			Tetraselmis suecica	4	FDA metabolic activity, chlorophyll fluorescence kinetics, chlorophyll *a* content	5[5]<br>2.5[2]<br>7.5[10]	9
		Cyanobacteria	*Microcystis aeruginosa*	11	Chlorophyll fluorescence kinetics (F_v/F_m) ,chlorophyll a and pheophytin content	4[2,4]	90

			9	The cell density, chlorophyll *a* and protein content, SOD, CAT, POD activities	0.05[5,9]<br>0.2[1]	52
			6	Microcystin synthesis	3.02[7](EC_{10})	88
			27	Microcystin synthesis	0.1-0.25	89
		Nodularia spumigena	11	Chlorophyll fluorescence kinetics (F_v/F_m), chlorophyll *a* and pheophytin content	0.1[5]<br>1[2]	90
	Diatom	*Phaeodactylum tricornutum*	11	Chlorophyll fluorescence kinetics (F_v/F_m), chlorophyll a and pheophytin content	1[5]<br>0.1[2]	90
TCN	Green algae	*Chlorella vulgaris*	4	Cell structure, SOD and CAT content	> 5[1]<br>< 2.5[8]<br>5[1]	49
		Dictyosphaerium pulchellum	11	Chlorophyll fluorescence kinetics, protein content	5[2,9]	71
		Micractinium pusillum	11	Chlorophyll fluorescence kinetics, protein content	30[9]<br>20[2]	71
		Raphidocelis subcapitata	7	Chlorophyll fluorescence kinetics (F_v/F_m), SOD, MDA content	>0.2[2]<br>1[1]	28
	Cyanobacteria	*Microcystis aeruginosa*	7	Chlorophyll fluorescence kinetics (F_v/F_m), SOD, MDA content	> 0.5[2]<br>0.1[1]	28
				Microcystin synthesis	0.63[7] (EC_{10})	88
			27	Microcystin synthesis	0.1-0.25	89
			9	The cell density, chlorophyll *a* and protein content, SOD, CAT, POD activities	0.05[5,9]<br>0.2[1]	52
TGC	Green algae	*Chlorella pyrenoidosa*	6	SOD, CAT activity, MDA content, chlorophyll content	2[1]	92
	Cyanobacteria	*Anabaena cylindrica*	6	SOD, CAT activity, MDA content, chlorophyll content	0.1[1]	92

Quinolones	CIP	Green algae	*Raphidocelis subcapitata*	4	CAD, SOD, GPX and GST activity, the ascorbate - glutathione cycle, the xanthophyll cycle	2.5 [1]<br>1.5 [3,4]	80
				5	SOD activity and LPO levels, cellular energy allocation (CEA),	0.62 mg/L (LOEC)	81
				4	Chlorophyll *a* and carotenoid content, photosynthetic rate, chlorophyll biosynthesis inhibition	>1.5[5] mg/L<br>1.0 [6]	83
	ENR	Green algae	*Chlorella vulgaris*	16	Chlorophyll fluorescence kinetics (F_v/F_m), SOD, CAT, MDA activity, chlorophyll a content	5[5] mg/L<br>20[2]<br>1[1]	86
				4	Chlorophyll *a*, carotenoid, and MDA content	20[1]<br>1-100[5] mg/L	65
				4	MDA and GSH contents, chlorophyll *a* content	0.021[1,5] mg/L	37
			Micractinium resseri	4	Chlorophyll *a*, carotenoid, and MDA content	20[1]<br>1-100[5] mg/L	65
			Scenedesmus obliquus	4	Chlorophyll *a*, carotenoid, and MDA content	20[1]<br>1-100[5] mg/L	65
			Scenedesmus obliquus UTEX 78	3	Chlorophyll *a* content, chlorophyll fluorescence kinetics	24.6[5] (EC_{10})<br>49.6[2] (EC_{10})	66
			Scenedesmus obliquus MPI	3	Chlorophyll *a* content, chlorophyll fluorescence kinetics	30.2[5] (EC_{10})<br>49.8[2] (EC_{10})	66
			Scenedesmus obliquus SAG276/3a	3	Chlorophyll *a* content, chlorophyll fluorescence kinetics	36.9[5] (EC_{10})<br>17.1[2] (EC_{10})	66
				4	SOD and MDA content, superoxide anion (O^{-}_{2}) generation rate, chlorophyll, carotenoids, and protein content	80[5]<br>48[1,9]	67

	Cyanobacteria	*Chrysosporum ovalisporum*	16	Chlorophyll fluorescence kinetics (F_v/F_m), SOD, CAT, MDA activity, chlorophyll a content	1[2]<br>50[1]	86
			11	Phycobiliproteins (PBPs) content, and alkaline phosphatase (ALP) activity, chlorophyll *a* content	1[5]	92
		Microcystis aeruginosa SAG 1785	3	Chlorophyll *a* content, chlorophyll fluorescence kinetics	0.008[2] (EC_{10})	66
		Microcystis aeruginosa PCC7820	3	Chlorophyll *a* content, chlorophyll fluorescence kinetics	0.021[5] (EC_{10})<br>0.007[2] (EC_{10})	66
		Microcystis aeruginosa V131	3	Chlorophyll *a* content, chlorophyll fluorescence kinetics	0.006[2] (EC_{10})	66
GFX	Cyanobacteria	*Microcystis aeruginosa*	4	Microcystin synthesis, chlorophyll fluorescence kinetics (F_v/F_m), chlorophyll *a* and carotenoid, ROS, and MDA content	0.02[5]<br>0.05[1,2]<br>0.01[7]	68
MXF	Cyanobacteria	*Microcystis aeruginosa*	4	Microcystin synthesis, chlorophyll fluorescence kinetics (F_v/F_m), chlorophyll *a* and carotenoid, ROS, and MDA content	0.05[1,,2,5]<br>0.01[7]	68
NOR	Green algae	*Chlorella vulgaris*	16	Chlorophyll fluorescence kinetics (F_v/F_m), SOD, CAT, MDA activity, chlorophyll *a* content	5[5]<br>50[1,2]	86
	Cyanobacteria	*Chrysosporum ovalisporum*	16	Chlorophyll fluorescence kinetics (F_v/F_m), SOD, CAT, MDA activity, chlorophyll *a* content	1[2]<br>5[1]	86
			11	Phycobiliproteins content, and alkaline phosphatase (ALP) activity, chlorophyll *a* content	1[5]	92
		Microcystis aeruginosa	1	GSH simulation, SOD activity, MDA content, chlorophyll *a* content	10[1,5]	75
OFX	Cyanobacteria	*Microcystis aeruginosa*	4	Chlorophyll *a* and carotenoids content, chlorophyll fluorescence kinetics (F_v/F_m)	0.1[2,5]	91

CAT-catalase, EC_{10} –median effect concentration (10%), FDA- fluorescein diacetate, GSH –glutathione, GST- glutathione-S-transferase, GPX- guaiacol glutathione peroxidase, LOEC- lowest observed effective concentration, LPO- lipid peroxidation MDA –malondialdehyde, POD- peroxidase, SOD- superoxide dismutase * – degradation products, 1- oxidative stress, 2 – photosynthetic activity, 3- xanthophyll cycle, 4- the ascorbate - glutathione cycle, 5- pigments content, 6- chlorophyll biosynthesis inhibition, 7- microcystins synthesis, 8- cell structure alterations, 9- protein content, 10- metabolic activity, 11- O_2 evolution

References

1. Eguchi K., Nagase H., Ozawa M., Endoh Y.S., Goto K., Hirata K., Miyamoto K., Yoshimura H., Evaluation of antimicrobial agents for veterinary use in the ecotoxicity test using microalgae. *Chemosphere*, **2004**, *57*:1733–1738, DOI:10.1016/j.chemosphere.2004.07.017
2. Magdaleno A., Saenz M.E., Juárez A.B., Moretton J., 2015, Effects of six antibiotics and their binary mixtures on growth of *Pseudokirchneriella subcapitata*. *Ecotoxicol. Environ. Saf.*, **2015**, *113*:72-78. DOI:10.1016/j.ecoenv.2014.11.021.
3. Straub J.O., Gysel D., Kastl U., Klemmer J., Sonderegger M., Studer M., Environmental risk assessment for ancillary substances in biotechnological production of pharmaceuticals. *Environ. Toxicol. Chem.*, **2012**, *31*: 681-687. https://doi.org/10.1002/etc.1733
4. Qian H., Li J., Pa, X., Sun Z., Ye C., Jin G. and Fu Z., Effects of streptomycin on growth of algae *Chlorella vulgaris* and *Microcystis aeruginosa*. *Environ. Toxicol.*,**2010**, *27*: 229-237. https://doi.org/10.1002/tox.20636
5. Van der Grinten E., Pikkemaat M.G., van den Brandhof E.J., Stroomberg G.J., Kraak M.H.S., Comparing the sensitivity of algal, cyanobacterial and bacterial bioassays to different groups of antibiotics. *Chemosphere*, **2010**, *80*:1–6, DOI: 10.1016/j.chemosphere.2010.04.011
6. Halling-Sørensen B., Algal toxicity of antibacterial agents used in intensive farming. *Chemosphere*, **2000,** *40(7)*:731-9. doi: 10.1016/s0045-6535(99)00445-2.
7. Lai H.T., Hou J.H., Su C.I., Chen C.L., Effects of chloramphenicol, florfenicol, and thiamphenicol on growth of algae *Chlorella pyrenoidosa*, *Isochrysis galbana*, and *Tetraselmis chui*. *Ecotoxicol. Environ. Saf.*, **2009** , *72(2)*:329-34. DOI: 10.1016/j.ecoenv.2008.03.005.
8. Lofrano G., Libralato G., Adinolfi R., Siciliano A., Iannece P., Guida M., Giugni M., Volpi Ghirardini A., Carotenuto M., Photocatalytic degradation of the antibiotic chloramphenicol and effluent toxicity effects. *Ecotoxicol Environ Saf.*, **2016**, *123*:65-71. DOI: 10.1016/j.ecoenv.2015.07.039.
9. Seoane M., Rioboo C., Herrero C., Cid Á. Toxicity induced by three antibiotics commonly used in aquaculture on the marine microalga *Tetraselmis suecica* (Kylin) Butch. *Mar Environ Res*. **2014**, *101*:1-7. DOI: 10.1016/j.marenvres.2014.07.011.
10. Campa-Córdova A.I., Luna A., Ascencio F., Cortes-Jacinto E., Caceres C., Effects of chloramphenicol, erythromycin, and furazolidone on growth of *Isochrysis galbana* and *Chaetoceros gracilis*. *Aquaculture*, **2006**, *260:*145-150. 10.1016/j.aquaculture.2006.06.014.
11. Fu L., Huang T., Wang S., Wang X., Su L., Li C., Zhao Y., Toxicity of 13 different antibiotics towards freshwater green algae *Pseudokirchneriella subcapitata* and their modes of action, *Chemosphere*, **2017**, *168*: 217-222. DOI: 10.1016/j.chemosphere.2016.10.043
12. Christensen A.M., Ingerslev F., Baun A., Ecotoxicity of mixtures of antibiotics used in aquacultures, *Environ. Toxicol. Chem.*,**2006,** *25 (8)*:2208-2215. DOI: 10.1897/05-415r.1
13. Wang M., Zhang Y., Guo P., Effect of florfenicol and thiamphenicol exposure on the photosynthesis and antioxidant system of *Microcystis flos-aquae*. *Aquat Toxicol*. **2017,** *186*:67-76. DOI: 10.1016/j.aquatox.2017.02.022.
14. Liu W., Ming Y., Huang Z., Li P., Impacts of florfenicol on marine diatom *Skeletonema costatum* through photosynthesis inhibition and oxidative damages. *Plant Physiol. Biochem.* **2012**, *60*:165-70. DOI:10.1016/j.plaphy.2012.08.009.
15. Ando T., Nagase H., Eguchi K., Hirooka T., Nakamura T., Miyamoto K., Hirata K., A novel method using cyanobacteria for ecotoxicity test of veterinary antimicrobial agents, *Environ. Toxicol. Chem.*, **2007**, *26(4):*601-606. https://doi.org/10.1897/06-195R.1
16. Aubakirova, B.N., Beisenova, R.R., Zhamangara, A.K., The effect of pharmaceutical ingredients to the growth of algae. News of the national academy of sciences of the republic of Kazakhstan. *Series Biol. Med.*, **2017**, *322*: 5-11
17. Andreozzi R., Caprio V., Ciniglia C., de Champdoré M., Giudice R.L., Marotta R., Zuccato E., Antibiotics in the Environment: Occurrence in Italian STPs, Fate, and Preliminary Assessment on Algal Toxicity of Amoxicillin, *Environ. Sci. Technol.*, **2004**, *38(24):* 6832–6838. https://doi.org/10.1021/es049509a

18. Zhong X., Zhu Y., Wang Y., Zhao Q., Huang H., Effects of three antibiotics on growth and antioxidant response of *Chlorella pyrenoidosa* and *Anabaena cylindrica*., *Ecotoxicol Environ Saf.*, **2021**, *211(5)*:111954. 10.1016/j.ecoenv.2021.111954.
19. Gonzalez-Pleiter M., Gonzalo S., Rodea-Palomares I., Leganes F., Rosal R., Boltes K., Marco E., Fernandez-Pinas F., Toxicity of five antibiotics and their mixtures towards photosynthetic aquatic organisms: Implications for environmental risk assessment. *Water Res.*, **2013**, *47*:2050–2064, DOI: 10.1016/j.watres.2013.01.020
20. Lützhøft H.C.H., Halling-Sørensen B., Jørgensen S.E., Algal toxicity of antibacterial agents applied in Danish fish farming, *Arch. Environ. Contam. Toxicol.*, **1999**, *36(1)*:1-6, DOI: 10.1007/s002449900435
21. Coors A., et al.,. Joint Effects of Pharmaceuticals and Chemicals Regulated Under REACH in Wastewater Treatment Plant Effluents Evaluating. Evaluating Concepts for a Risk Assessment by Means of Experimental Scenarios. Dessau-Roßlau, Germany. **2017**
22. Liu Y., Gao B., Yue Q., Guan Y., Wang Y., Huang L., Influences of two antibiotic contaminants on the production, release and toxicity of microcystins. *Ecotoxicol. Environ. Saf.*, **2012**, *77*:79-87. DOI: 10.1016/j.ecoenv.2011.10.027.
23. Gao L., Shi L.J., Yuan T., Growth inhibitive effect of typical antibiotics and their mixtures on *Selenastrum capricornutum. J. Environ. Health*, **2013,** *30*:475-478
24. Borecka M., Białk-Bielińska A., Haliński Ł.P., Pazdro K., Stepnowski P., Stolte S., The influence of salinity on the toxicity of selected sulfonamides and trimethoprim towards the green algae *Chlorella vulgaris, J. Hazard. Mater*, **2016**, *308*:179-186, https://doi.org/10.1016/j.jhazmat.2016.01.041
25. Guo J., Selby K., Boxall A.B., Effects of Antibiotics on the Growth and Physiology of Chlorophytes, Cyanobacteria, and a Diatom., *Arch Environ Contam Toxicol.*, **2016**, *71(4):*589-602. DOI: 10.1007/s00244-016-0305-5.
26. De Liguoro M., Di Leva V., Dalla Bona M., Merlanti R., Caporale G., Radaelli G., Sublethal effects of trimethoprim on four freshwater organisms., *Ecotoxicol Environ Saf.*, **2012**, *82*:114-21. DOI: 10.1016/j.ecoenv.2012.05.016.
27. Kolar B., Arnus L., Jeretin B., Gutmaher A., Drobne D., Durjava M.K., The toxic effect of oxytetracycline and trimethoprim in the aquatic environment. *Chemosphere,* **2014**, *115*: 75–80, DOI: 10.1016/j.chemosphere.2014.02.049
28. Yang L.H., Ying G.G., Su H.C., Stauber J.L., Adams M.S., Binet M.T., Growth-inhibiting effects of 12 antibacterial agents and their mixtures on the freshwater microalga *Pseudokirchneriella subcapitata. Environ Toxicol Chem.* **2008,** *27(5):*1201-8. DOI: 10.1897/07-471.1
29. Claessens M., Vanhaecke L., Wille K., Janssen C.R. Emerging contaminants in Belgian marine waters: single toxicant and mixture risks of pharmaceuticals. *Mar. Pollut. Bull.* **2013**, *71(1-2):*41-50. DOI: 10.1016/j.marpolbul.2013.03.039.
30. Villain J., Minguez L., Halm-Lemeille M.P., Durrieu G., Bureau R. Acute toxicities of pharmaceuticals toward green algae. mode of action, biopharmaceutical drug disposition classification system and quantile regression models. *Ecotoxicol. Environ Saf.,* **2016,** *124*:337-343. DOI: 10.1016/j.ecoenv.2015.11.009.
31. Isidori M., Lavorgna M., Nardelli A., Pascarella L., Parrella A., Toxic and genotoxic evaluation of six antibiotics on non-target organisms, *Sci. Total Environ.* , **2005**, *346*: 87-98. DOI: 10.1016/j.scitotenv.2004.11.017
32. Hagenbuch I.M., Pinckney J.L., Toxic effect of the combined antibiotics ciprofloxacin, lincomycin, and tylosin on two species of marine diatoms. *Water Res.*, **2012**, *46(16):*5028-36. DOI: 10.1016/j.watres.2012.06.040.
33. Harada A., Komori K., Nakada N., Kitamura K., Suzuki Y.; Biological effects of PPCPs on aquatic lives and evaluation of river waters affected by different wastewater treatment levels. *Water Sci. Technol.*, **2008**, *58 (8):* 1541–1546. DOI: https://doi.org/10.2166/wst.2008.742
34. Baumann M., Weiss K., Maletzki D., Schüssler W., Schudoma D., Kopf W., Kühnen U. Aquatic toxicity of the macrolide antibiotic clarithromycin and its metabolites. *Chemosphere.* **2015**, *120*:192-8. DOI: 10.1016/j.chemosphere.2014.05.089.
35. Watanabe H., Tamura I., Abe R., Takanobu H., Nakamura A., Suzuki T., Hirose A., Nishimura T., Tatarazako N., Chronic toxicity of an environmentally relevant mixture of pharmaceuticals to three aquatic organisms (alga, daphnia, and fish). *Environ Toxicol Chem.* **2016**, *35(4):*996-1006. DOI: 10.1002/etc.3285.

36. Yamashita N., Yasojima M., Nakada N., Miyajima K., Komori K., Suzuki Y., Tanaka H. Effects of antibacterial agents, levofloxacin and clarithromycin, on aquatic organisms. *Water Sci. Technol.,* **2006**, *53(11):*65-72. DOI: 10.2166/wst.2006.338.
37. Wang G., Zhang Q., Li J., Chen X., Lang Q., Kuang S., Combined effects of erythromycin and enrofloxacin on antioxidant enzymes and photosynthesis-related gene transcription in *Chlorella vulgaris*., *Aquatic Toxicology*, **2019,** *21*:138-145, https://doi.org/10.1016/j.aquatox.2019.05.004.
38. Sendra M., Moreno-Garrido I., Blasco J., Araújo C., Effect of erythromycin and modulating effect of CeO_2 NPs on the toxicity exerted by the antibiotic on the microalgae *Chlamydomonas reinhardtii* and *Phaeodactylum tricornutum*. *Environ. Pollut.,* **2018**, *242*: 357-366. DOI: 10.1016/j.envpol.2018.07.009.
39. Wu, Y.; Wan, L.; Zhang, W.; Ding, H.; Yang, W. Resistance of cyanobacteria *Microcystis aeruginosa* to erythromycin with multiple exposure. *Chemosphere*, **2020,** *249*:126147. https://doi.org/10.1016/j.chemosphere.2020.126147
40. Lanzky P.F., Halling-Sørensen B., The toxic effect of the antibiotic metronidazole on aquatic organisms. *Chemosphere.* **1997**, *35(11):*2553-61. DOI: 10.1016/s0045-6535(97)00324-x.
41. Białk-Bielińska A., Stolte S., Arning J., Uebers U., Böschen A., Stepnowski P., Matzke M., Ecotoxicity evaluation of selected sulfonamides, *Chemosphere*, **2011**, *85(6):*928-933
42. De Orte M. R., Carballeira C., Viana I. G., Carballeira A., Assessing the toxicity of chemical compounds associated with marine land-based fish farms: The use of mini-scale microalgal toxicity tests, *Chemistry and Ecology*, **2013**, *29(6)*: 554-563, DOI:10.1080/02757540.2013.790381
43. Pomati F., Netting A.G., Calamari D., Neilan B.A. Effects of erythromycin, tetracycline and ibuprofen on the growth of *Synechocystis* sp. and *Lemna minor. Aquat Toxicol.* **2004,** *12:67(4):*387-96. DOI: 10.1016/j.aquatox.2004.02.001.
44. Li J., Min Z., Li W., Interactive effects of roxithromycin and freshwater microalgae, *Chlorella pyrenoidosa*: Toxicity and removal mechanism. *Ecotoxicol Environ Saf.* **2020**, *191*:110156. DOI: 10.1016/j.ecoenv.2019.110156.
45. Wang Z., Chen Q., Hu L., Wang M., Combined effects of binary antibiotic mixture on growth, microcystin production, and extracellular release of *Microcystis aeruginosa*: application of response surface methodology., *Environ Sci. Pollut Res.,* **2018**, *25*:736–748. https://doi.org/10.1007/s11356-017-0475-3
46. Xiong, J. Q., Govindwar, S., Kurade, M. B., Paeng, K. J., Roh, H. S., Khan, M. A., & Jeon, B. H., Toxicity of sulfamethazine and sulfamethoxazole and their removal by a green microalga, *Scenedesmus obliquus*. *Chemosphere*, **2019**, *218*:551-558. https://doi.org/10.1016/j.chemosphere.2018.11.146
47. Baran W., Sochacka J., Wardas W., Toxicity and biodegradability of sulfonamides and products of their photocatalytic degradation in aqueous solutions, *Chemosphere*, **2006**, *65*:1295-1299. DOI: 10.1016/j.chemosphere.2006.04.040
48. Ferrari B., Mons R., Vollat B., Fraysse B., Paxēaus N., Giudice R.L, Environmental risk assessment of six human pharmaceuticals: are the current environmental risk assessment procedures sufficient for the protection of the aquatic environment, *Environ. Toxicol. Chem*., **2004**, *23(5):* 1344-1354. DOI: 10.1897/03-246
49. Xu D., Xiao Y., Pan H., Mei Y., Toxic effects of tetracycline and its degradation products on freshwater green algae., *Ecotoxicol Environ Saf.* **2019**, *15*:174:43-47. DOI: 10.1016/j.ecoenv.2019.02.063
50. Carusso S., Juárez A.B., Moretton J., Magdaleno A., Effects of three veterinary antibiotics and their binary mixtures on two green alga species *Chemosphere*,**2018**, *194*:821-827. DOI: 10.1016/j.chemosphere.2017.12.047
51. Lu L., Wu Y., Ding H., Zhang W., The combined and second exposure effect of copper (II) and chlortetracycline on fresh water algae, *Chlorella pyrenoidosa* and *Microcystis aeruginosa*, *Environ Toxicol. Pharmacol.,* **2015**, *40(1):*140-8. DOI: 10.1016/j.etap.2015.06.006.
52. Shang A.H., Ye J., Chen D.H., Lu X.X., Lu H.D., Liu C.N., Wang L.M., Physiological effects of tetracycline antibiotic pollutants on non-target aquatic *Microcystis aeruginosa. J Environ Sci. Health B.,* **2015**, *50(11):*809-18. DOI: 10.1080/03601234.2015.1058100.
53. Stoichev T., Baptista M.S., Basto M.C., Vasconcelos V.M., Vasconcelos M.T., Effects of minocycline and its degradation products on the growth of *Microcystis aeruginosa*. *Ecotoxicol Environ Saf.,* **2011,** *74(3):*219-24. DOI: 10.1016/j.ecoenv.2010.10.015.

54. Magdaleno A., Carusso S., Moretton, , Toxicity and Genotoxicity of Three Antimicrobials Commonly Used in Veterinary Medicine. *J. Bull. Environ. Toxicol.*, **2017**, *99*: 315. DOI: 10.1007/s00128-017-2091-9
55. Pro J., Ortiz J.A., Boleas S., Fernández C., Carbonell G., Tarazona J.V., , Effect assessment of antimicrobial pharmaceuticals on the aquatic plant *Lemna minor, Bull. Environ. Contam. Toxicol.,* **2003**, 70:290–295. DOI: 10.1007/s00128-002-0208-1
56. Zounková, R., Klimešová, Z., Nepejchalová, L., Hilscherová, K. and Bláha, L, Complex evaluation of ecotoxicity and genotoxicity of antimicrobials oxytetracycline and flumequine used in aquaculture. *Environ. Toxicol. Chem.*, **2011**, *30*: 1184-1189. https://doi.org/10.1002/etc.486
57. Yang W., Tang Z., Zhou F., Zhang W., Song L., Toxicity studies of tetracycline on *Microcystis aeruginosa* and *Selenastrum capricornutum.*, *Environ Toxicol Pharmacol.* **2013**, *35(2)*:320-4. DOI: 10.1016/j.etap.2013.01.006.
58. Geiger E., Hornek-Gausterer R., Saçan M.T. Single and mixture toxicity of pharmaceuticals and chlorophenols to freshwater algae *Chlorella vulgaris*., *Ecotoxicol. Environ. Saf.,* **2016**, *129:*189-98. DOI: 10.1016/j.ecoenv.2016.03.032.
59. Nie X., Wang X., Chen J., Taichen Z. V., An Response of the freshwater alga *Chlorella vulgaris* to trichloroisocyanuric acid and ciprofloxacin, *Environ. Toxicol. Chem.,* **2008,** *27*:168-173
60. Ebert I., Bachmann J., Kühnen U., Küster A., Kussatz C., Maletzki D, Schlüter C., Toxicity of the fluoroquinolone antibiotics enrofloxacin and ciprofloxacin to photoautotrophic aquatic organisms., *Environ. Toxico.l Chem*., **2011**, *30(12):*2786-92. DOI: 10.1002/etc.678.
61. Zhu L., Santiago-Schübel B., Xiao H., Hollert H., Kueppers S., Electrochemical oxidation of fluoroquinolone antibiotics: Mechanism, residual antibacterial activity and toxicity change. *Water Res.,* **2016,** *102*:52-62. DOI: 10.1016/j.watres.2016.06.005.
62. Robinson A.A., Belden J.B., Lydy M.J. Toxicity of fluoroquinolone antibiotics to aquatic organisms., *Environ Toxicol Chem*., **2005**, *24(2):*423-30. DOI: 10.1897/04-210r.1.
63. Martins N., Pereira R., Abrantes N., Pereira J., Gonçalves F., Marques C.R. Ecotoxicological effects of ciprofloxacin on freshwater species: data integration and derivation of toxicity thresholds for risk assessment., *Ecotoxicology*, **2012**, *21(4):*1167-76. DOI: 10.1007/s10646-012-0871-x.
64. Azevedo F. C., Rezende Vaz I. C. D., Barbosa F., Antônio R., Magalhães Sérgia M. S. Toxicological effects of ciprofloxacin and chlorhexidine on growth and chlorophyll a synthesis of freshwater cyanobacteria. B. J. Pharma. Sci. **2019**, *55*:7661. .https://doi.org/10.1590/s2175-97902019000217661
65. Xiong J.Q., Kurade M.B., Jeon B.H., Ecotoxicological effects of enrofloxacin and its removal by monoculture of microalgal species and their consortium., *Environ Pollut.* **2017** , *226:*486-493. DOI: 10.1016/j.envpol.2017.04.044.
66. Rico A., Zhao W., Gillissen F., Lürling M., Van den Brink P.J. Effects of temperature, genetic variation and species competition on the sensitivity of algae populations to the antibiotic enrofloxacin. *Ecotoxicol. Environ. Saf.* **2018**, *148:*228-236. DOI: 10.1016/j.ecoenv.2017.10.010.
67. Qin H., Chen L., Lu N., Toxic effects of enrofloxacin on *Scenedesmus obliquus*. *Front. Environ. Sci. Eng*., 2012, *6:*107–116. https://doi.org/10.1007/s11783-011-0327-1
68. Wan L., Wu Y., Zhang B., Yang W., Ding H., Zhang W. Effects of moxifloxacin and gatifloxacin stress on growth, photosynthesis, antioxidant responses, and microcystin release in *Microcystis aeruginosa*. *J Hazard Mater*. **2020**, *9:*124518. DOI: 10.1016/j.jhazmat.2020.124518.
69. Wan J., Guo P., Zhang S., Response of the cyanobacterium *Microcystis flos-aquae* to levofloxacin. *Environ Sci Pollut Res,* **2014**, 21:3858–3865, https://doi.org/10.1007/s11356-013-2340-3
70. Cameron J.C., Pakrasi H.B. Glutathione facilitates antibiotic resistance and photosystem I stability during exposure to gentamicin in cyanobacteria., *Appl Environ Microbiol.*, **2011**, *77(10):*3547-50. DOI: 10.1128/AEM.02542-10.
71. Bashir K.M., Cho M.G., The Effect of Kanamycin and Tetracycline on Growth and Photosynthetic Activity of Two Chlorophyte Algae., *Biomed Res Int*. **2016**, *2016:*5656304. DOI: 10.1155/2016/5656304.
72. Kvíderová J., Henley W.J. The effect of ampicillin plus streptomycin on growth and photosynthesis of two halotolerant chlorophyte algae. *J Appl Phycol,* **2005**, 17:301–307. https://doi.org/10.1007/s10811-005-7293-6
73. Liu Y., Guan Y., Gao B., Yue Q. Antioxidant responses and degradation of two antibiotic contaminants in *Microcystis aeruginosa*. *Ecotoxicol Environ Saf.,* **2012**, *86*:23-30. DOI: 10.1016/j.ecoenv.2012.09.004

74. Liu Y., Zhang J., Gao B., Feng S., Combined effects of two antibiotic contaminants on *Microcystis aeruginosa*. *J Hazard Mater.*, **2014**, *30:*279:148-55. DOI: 10.1016/j.jhazmat.2014.07.002.
75. Du Y., Wang J., Zhu F., Mai D., Xiang Z., Chen J., Guo R. Comprehensive assessment of three typical antibiotics on cyanobacteria (*Microcystis aeruginosa*): The impact and recovery capability. *Ecotoxicol Environ Saf.*, **2018,** *30:*160:84-93. DOI: 10.1016/j.ecoenv.2018.05.035.
76. Pan X., Deng C., Zhang D., Wang J., Mu G., Chen Y., , Toxic effects of amoxicillin on the photosystem II of Synechocystis sp. characterized by a variety of in vivo chlorophyll fluorescence tests, *Aquatic Toxicology,* **2008**, *89(4):*207-213, DOI: 10.1016/j.aquatox.2008.06.018
77. Wang Z., Chen Q., Zhang J., Dong J., Ao Y., Wang M., Wang X., Long-term exposure to antibiotic mixtures favors microcystin synthesis and release in *Microcystis aeruginosa* with different morphologies. *Chemosphere*, **2019**, *235*:344-353. DOI: 10.1016/j.chemosphere.2019.06.192.
78. Chen J.Q., Guo R.X. Access the toxic effect of the antibiotic cefradine and its UV light degradation products on two freshwater algae. *J Hazard Mater.*, **2012,** *30:*209-210:520-3. DOI: 10.1016/j.jhazmat.2012.01.041.
79. Guo J., Peng J., Lei Y., Kanerva M., Li Q., Song J., Guo J., Sun H. Comparison of oxidative stress induced by clarithromycin in two freshwater microalgae *Raphidocelis subcapitata* and *Chlorella vulgaris*. *Aquat Toxicol*. **2020**, *219:*105376. DOI: 10.1016/j.aquatox.2019.105376.
80. Nie X. P., Liu B. Y., Yu H., Liu W., Yang Y., Toxic effects of erythromycin, ciprofloxacin, and sulfamethoxazole exposure to the antioxidant system in *Pseudokirchneriella subcapitata*, *Environ. Pollution* ,**2013**, *172:* 23-32. DOI: 10.1016/j.envpol.2012.08.013
81. Aderemi A.O., Novais S.C., Lemos M.F.L., Alves L.M, Hunter C., Pahl O. Oxidative stress responses and cellular energy allocation changes in microalgae following exposure to widely used human antibiotics. *Aquat Toxicol.* **2018**, *203*:130-139. DOI: 10.1016/j.aquatox.2018.08.008.
82. Machado M., Soares E., Impact of erythromycin on a non-target organism: Cellular effects on the freshwater microalga *Pseudokirchneriella subcapitata*. *Aquatic Toxicology, **2019**, 208:*179–186, https://doi.org/10.1016/j.aquatox.2019.01.014
83. Liu B.Y., Nie X.P., Liu W.Q., Snoeijs P., Guan C., Tsui M.T. Toxic effects of erythromycin, ciprofloxacin and sulfamethoxazole on photosynthetic apparatus in *Selenastrum capricornutum*. *Ecotoxicol Environ Saf.* **2011**, *74(4):*1027-35. DOI: 10.1016/j.ecoenv.2011.01.022
84. Zhang M., Steinman A.D., Xue Q., Zhao Y., Xu Y., Xie L. Effects of erythromycin and sulfamethoxazole on *Microcystis aeruginosa*: Cytotoxic endpoints, production and release of microcystin-LR. *J Hazard Mater*. **2020**, *15*:399:123021. DOI 10.1016/j.jhazmat.2020.123021.
85. Wan J., Guo P., Peng X., Wen K. Effect of erythromycin exposure on the growth, antioxidant system and photosynthesis of *Microcystis flos-aquae*. *J Hazard Mater.* **2015,** 283:778-86. DOI: 10.1016/j.jhazmat.2014.10.026.
86. Chen S., Zhang W., Li J., Yuan M., Zhang J., Xu F., Xu H., Zheng X., Wang L. Ecotoxicological effects of sulfonamides and fluoroquinolones and their removal by a green alga (*Chlorella vulgaris*) and a cyanobacterium (*Chrysosporum ovalisporum). Environ Pollut*. **2020**, *263*:114554. DOI: 10.1016/j.envpol.2020.114554.
87. Guo R.X., Chen J.Q. Phytoplankton toxicity of the antibiotic chlortetracycline and its UV light degradation products. *Chemosphere.* **2012** , *87(11*):1254-9. DOI: 10.1016/j.chemosphere.2012.01.031
88. Ye J., Du Y., Wang L., Qian J., Chen J., Wu Q., Hu X. Toxin Release of Cyanobacterium *Microcystis aeruginosa* after Exposure to Typical Tetracycline Antibiotic Contaminants. *Toxins (Basel).* **2017**, *21:9(2):*53. DOI: 10.3390/toxins9020053.
89. Ye J., Huang C., Shang A., Xu C., Wu L. Characteristics of toxin production and release in *Microcystis aeruginosa* exposed to three tetracycline antibiotics. *Environ Sci Pollut Res Int.* **2020**, *27(14):*16798-16805. DOI: 10.1007/s11356-020-08253-x.
90. Siedlewicz G, Żak A., Sharma L., Kosakowska A, Pazdro K., Effects of oxytetracycline on growth and chlorophyll a fluorescence in green algae (*Chlorella vulgaris*), diatom (*Phaeodactylum tricornutum*) and cyanobacteria (*Microcystis aeruginosa* and *Nodularia spumigena*), *Oceanologia, **2020**, 62(2):* 214-225.
91. Deng C., Pan X., Zhang D. Influence of ofloxacin on photosystems I and II activities of *Microcystis aeruginosa* and the potential role of cyclic electron flow. *J Biosci. Bioeng*. **2015,** *119(2):*159-64. DOI: 10.1016/j.jbiosc.2014.07.014.

92. Zhang M., Steinman A.D., Xue Q., Zhao Y., Xu Y., Xie L. Effects of erythromycin and sulfamethoxazole on Microcystis aeruginosa: Cytotoxic endpoints, production and release of microcystin-LR. *J Hazard Mater*. **2020**, *15*:399:123021. DOI 10.1016/j.jhazmat.2020.123021.
93. Liu J. Y. Li, X. Yang, Y. T. , Nie X. P. Toxic effects of nutylated hydroxyanisole and norfloxacin on aquatic organisms. *Ecol. Sci.,* **2007**, *26:55*–58
94. Wu Y., Wan L., Zhang W., Ding H., Yang W. Resistance of cyanobacteria Microcystis aeruginosa to erythromycin with multiple exposure. *Chemosphere.* **2020,** *249*:126147. DOI: 10.1016/j.chemosphere.2020.126147.
95. Halling-Sørensen B., Lützhøft H.C., Andersen H.R., Ingerslev F., Environmental risk assessment of antibiotics: comparison of mecillinam, trimethoprim and ciprofloxacin., *J Antimicrob Chemother*., **2000**, *46(1):*53-8
